# The impact of co-exposure to air and noise pollution on the incidence of metabolic syndrome from a health checkup cohort

**DOI:** 10.1038/s41598-024-59576-5

**Published:** 2024-04-17

**Authors:** Jia-Hong Tang, Hong-Lian Jian, Ta-Chien Chan

**Affiliations:** 1 Research Center for Humanities and Social Sciences, Academia Sinica, 128 Academia Road, Section 2, Nankang, Taipei, 115 Taiwan; 2 Institute of Public Health, School of Medicine, National Yang Ming Chiao Tung University, Taipei, Taiwan; 3 Department of Public Health, College of Public Health, China Medical University, Taichung, Taiwan; 4 School of Medicine, College of Medicine, National Sun Yat-Sen University, Kaohsiung, Taiwan

**Keywords:** Metabolic syndrome, Air pollution, Noise, Environmental exposure, Chronic disease, Health impact, Environmental impact, Epidemiology

## Abstract

Previous studies have found associations between the incidence of metabolic syndrome (MetS) and exposure to air pollution or road traffic noise. However, investigations on environmental co-exposures are limited. This study aimed to investigate the association between co-exposure to air pollution and road traffic noise and MetS and its subcomponents. Participants living in Taipei City who underwent at least two health checkups between 2010 and 2016 were included in the study. Data were sourced from the MJ Health database, a longitudinal, large-scale cohort in Taiwan. The monthly traffic noise exposure (L_den_ and L_night_) was computed using a dynamic noise map. Monthly fine particulate data at one kilometer resolution were computed from satellite imagery data. Cox proportional hazards regression models with month as the underlying time scale were used to estimate hazard ratios (HRs) for the impact of PM_2.5_ and road traffic noise exposure on the risk of developing MetS or its subcomponents. Data from 10,773 participants were included. We found significant positive associations between incident MetS and PM_2.5_ (HR: 1.88; 95% CI 1.67, 2.12), L_den_ (HR: 1.10; 95% CI 1.06, 1.15), and L_night_ (HR: 1.07; 95% CI 1.02, 1.13) in single exposure models. Results further showed significant associations with an elevated risk of incident MetS in co-exposure models, with HRs of 1.91 (95% CI 1.69, 2.16) and 1.11 (95% CI 1.06, 1.16) for co-exposure to PM_2.5_ and L_den_, and 1.90 (95% CI 1.68, 2.14) and 1.08 (95% CI 1.02, 1.13) for co-exposure to PM_2.5_ and L_night_. The HRs for the co-exposure models were higher than those for models with only a single exposure. This study provides evidence that PM_2.5_ and noise exposure may elevate the risk of incident MetS and its components in both single and co-exposure models. Therefore, preventive approaches to mitigate the risk of MetS and its subcomponents should consider reducing exposure to PM_2.5_ and noise pollution.

## Introduction

Metabolic syndrome (MetS) is a global public health concern because it increases the risk of many chronic diseases, including cardiovascular disease (CVD), type-2 diabetes, hypertension, and certain cancers. MetS is defined as a cluster of the following five conditions: abdominal obesity, high blood pressure, impaired fasting glucose, high triglycerides, and low high-density lipoprotein (HDL) cholesterol. It is diagnosed when at least three of these conditions are present. Metabolic and behavioral risk factors, including age, sex, genetics, race, ethnicity, and lifestyle factors, such as physical activity and socioeconomic status, are the most common causes of MetS^1–3^.

Environmental pollution dates back to the onset of urbanization. While urbanization has benefits, such as improved employment opportunities, quality of life, and access to higher education and health services, it also has disadvantages, such as increased exposure to various environmental pollutants. Environmental pollution results directly from intensive traffic, construction, and industrial activity. These factors can combine and sometimes act synergistically to cause vascular dysfunction and metabolic abnormalities, critically affecting the health and well-being of city residents^4^.

Air and noise pollution are the most common environmental risk factors that residents encounter in urban areas and have been linked to metabolic disorders that are prodromal to many chronic diseases^5–7^. Air pollution, which is considered the largest single environmental risk factor for pollution-related adverse health outcomes, has drawn significant public health concern globally^8,9^. More than 80% of the world’s population lives in areas where particulate levels equal or exceed the thresholds recommended by the World Health Organization^10^. Previous studies have indicated that long-term exposure to ambient air pollutants, including PM_2.5_, could increase the risk of MetS^11–13^. Increased exposure to PM_2.5_ has also been found to contribute to increased incidences or prevalence of obesity^14,15^, diabetes^16–18^, hypertension^19–21^, dyslipidemia^22,23^ and low HDL^24,25^.

Noise is the second most significant environmental threat to health, with exposure to environmental noise causing 12,000 premature deaths annually and contributing to 48,000 new cases of ischemic heart disease in Europe^26^. Several large-scale cohort studies have shown that long-term exposure to excessive noise influences environmental changes and the quality of life or health of human beings. A nationwide cohort study in Switzerland found that traffic noise may increase the hazard risk of mortality by approximately 2–5%, and also observed elevated risks related to CVD, heart failure, blood pressure, ischemic stroke, and myocardial infarction^27^. They also investigated the diurnal variability of transportation noise and mortality and reported the highest hazard ratio for core night hours (1:00–5:00) and ischemic heart disease mortality, suggesting that nighttime noise exposure is very important in relation to CVD risk^28^. Their recent study also found that the association between transportation noise and death from myocardial infarction in a Swiss population was stable after adjusting for air pollution^29^. Three large Danish cohort studies on traffic-related pollution and health examined the effects of PM_2.5_, NO_2_ and traffic noise and observed elevated risks of CVD, heart failure, blood pressure, ischemic stroke, myocardial infarction mortality^30^, type 2 diabetes^31^ and atrial fibrillation^32^.

In Europe, air pollution, together with traffic noise pollution contribute to more than 75% of the burden of disease attributable to environmental factors^33^. People living in cities are exposed to multiple environmental factors simultaneously, which may impact health in combination and, in some cases, act synergistically. Many studies have investigated the effect of a single environmental pollutant on MetS or one of its subcomponents; however, evidence concerning the effects of co-exposure to air and noise pollution on MetS and its subcomponents remains scarce. This study investigated the effects of single and co-exposure to air pollution and road traffic noise on the incidence of MetS and its components in a health checkup cohort of participants living in Taipei City, Taiwan. Fine-scale temporal resolution was used to enhance our understanding of the development of MetS and its subcomponents. Additionally, we evaluated the potential confounding effects of basic information, personal and family health history, lifestyle and behavior, physical activity, sleep duration, and dietary habits.

## Materials and methods

### Study area, design, and participants

This study was a retrospective cohort study. We investigated the impact of previous environmental exposure on the risk of developing MetS and its components. Participant data was collected from the MJ Health Database (MJHD) to include 10,997 Taipei City residents who underwent at least two health checkups at the MJ Health Management Institution, a private healthcare firm in Taiwan, between 2010 and 2016. The MJ Health Database is a longitudinal, large-scale, comprehensive population-based health database. For each examination visit, information on behavior and lifestyle was collected via a self-administered health questionnaire. Data on anthropometric and biological tests were collected via health checkups.

**Figure 1:**
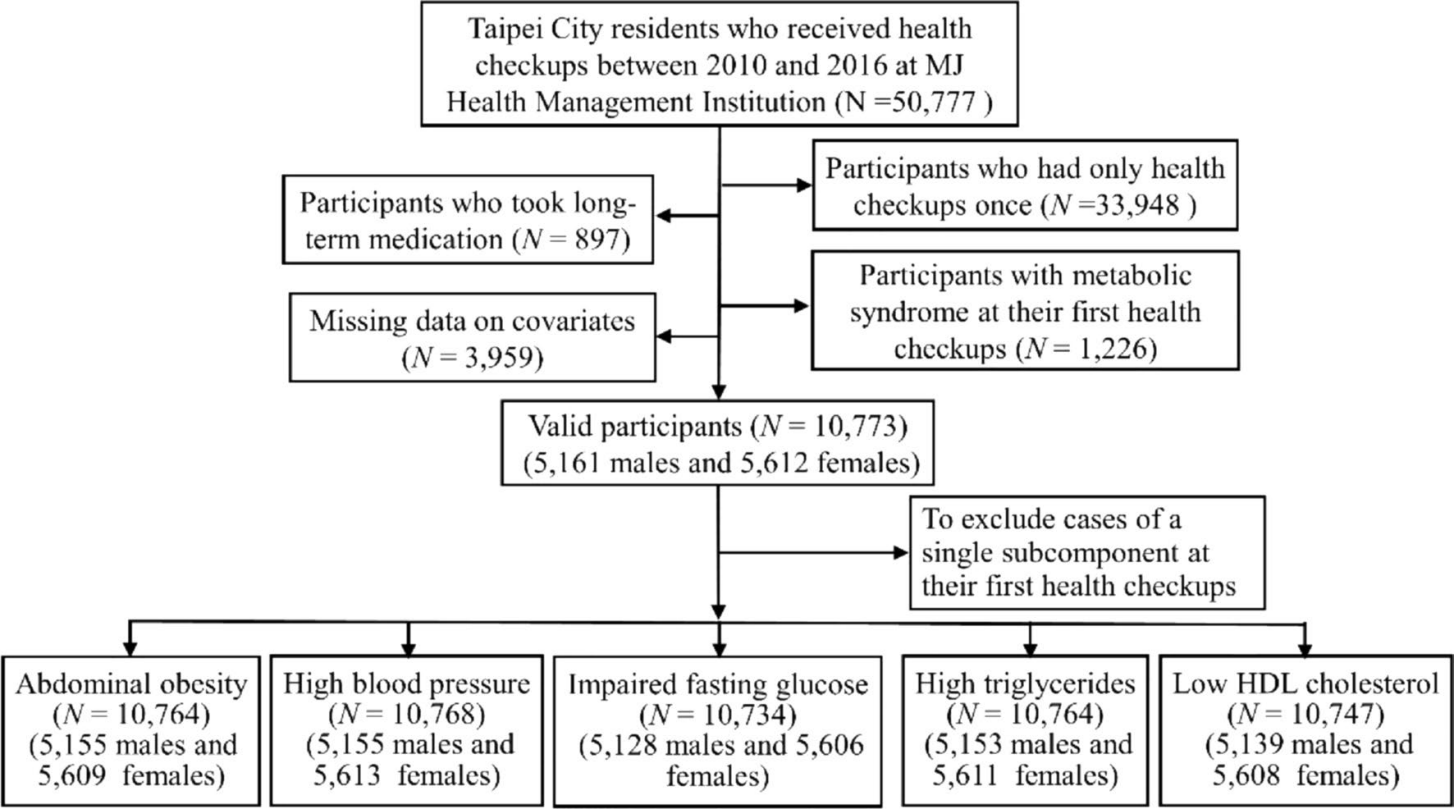
The flowchart of participants’ selection. The flowchart shows the exclusion criteria for participant selection and shows the final number of participants included (N = 10,773 and their distribution across the different subcomponents of the metabolic syndrome).

Figure 1 shows a flowchart of the data-selection process. The initial study participants included 50,777 individuals who underwent health check-ups between 2010 and 2016. Participants were eligible if they did not develop MetS during the first examination. We excluded participants who (1) had only received one health checkup (*N* = 33,948), (2) had missing data on outcome and confounding variables (*N* = 3959), (3) took long-term medication (*N* = 897), and (4) had incident MetS at their first visit (*N* = 1226); the remaining 10,773 participants were enrolled and followed up for MetS.

To further assess the risk of environmental exposure for each subcomponent of MetS, the respective baseline sample for each subcomponent was composed of the data from valid participants who were tested negative for that subcomponent at their first health checkup. Therefore, we included *N* = 10,764 for abdominal obesity, *N* = 10,768 for high blood pressure, *N* = 10,734 for impaired fasting glucose, *N* = 10,764 for high triglycerides, and *N* = 10,747 for low HDL cholesterol. The follow-up period was from the date of their previous health checkup to the date of the health checkup for MetS or a single subcomponent. For participants without MetS or any single subcomponent, the follow-up period ranged from the date of their first health checkup to the date of their last health checkup.

The geocoding process was handled by the MJ Health Resource Center before being released to the data users. They used two geocoders to geocode the addresses from health check-up visits, including the official geocoder, TGOS (https://www.tgos.tw/tgos/Addr), operated by the Ministry of Interior, Taiwan, and the Google Map geocoding service (https://developers.google.com/maps/documentation/geocoding). Our research team used the released coordinates to link exposure to noise and PM_2.5_.

### Outcome definition

According to the Health Promotion Administration, Ministry of Health and Welfare, Taiwan, metabolic syndrome in Taiwan is diagnosed with the presence of at least three of the following five criteria: (1) abdominal obesity: waist circumference ≥ 90 cm in men and ≥ 80 cm in women (2) high blood pressure: systolic blood pressure ≥ 130 mmHg or diastolic blood pressure ≥ 85 mmHg (3) impaired fasting glucose: fasting plasma glucose ≥ 100 mg/dL (4) high triglycerides: triglycerides ≥ 150 mg/dL (5) low HDL cholesterol: HDL cholesterol < 40 mg/dL in men and < 50 mg/dL in women^34^.

Venipuncture was performed to collect overnight fasting blood for a series of biochemical analyses, including lipid profile, renal function, liver profile, and serum albumin, globulin, and uric acid levels. All biological specimens were analyzed in a laboratory located at the MJ Health Screening Center. A standardized service workflow was adopted by all four health-screening centers in Taiwan that conformed to ISO 9001-2008 requirements for quality management systems. The MJ Health Screening Center laboratories participated in College of American Pathologists Proficiency Testing to ensure their ability to generate good-quality health data.

### Exposure measurement

Personal PM_2.5_ exposures were assessed using high-resolution, specified air pollution datasets maintained by the Atmospheric Composition Analysis Group of Washington University in St. Louis. These were global estimates of ground-level ambient PM_2.5_, which were gridded at approximately 1 km × 1 km^35^. The PM_2.5_ concentrations were available at a monthly timescale for the years 2010 through 2016. Briefly, PM_2.5_ was estimated based on several satellite aerosol optical depth (AOD) products, and then combined with chemical transport model simulations to relate AOD to the estimated surface-level PM_2.5_ concentrations. The estimated monthly PM_2.5_ concentrations were validated against ground PM_2.5_ concentrations measured at fixed monitoring stations across Taipei City. The PM_2.5_ estimates exhibited general consistency with ground-based observations, with a Pearson correlation coefficient of 0.69. The model accuracy was evaluated by mean absolute error (MAE) = 4.31 µg/m^3^, and root mean square error (RMSE) = 5.42 µg/m^3^.

Noise exposure was assessed based on road traffic noise levels. The hourly noise levels for road segments were estimated using noise prediction models with traffic conditions and road attributes as inputs^36^. Historical traffic condition data collected by available overhead microwave vehicle detectors in Taipei City were provided by the Department of Transportation, Taipei City Government. The geographical data included road network and building data in a vector format. Information on the road segments includes the following attributes: road identification (ID), road name, length, width, number of lanes, and road class. A-weighted sound pressure levels in decibels (dBA) were estimated over 24 h. We assumed that the hourly noise levels of each participant depended mainly on the noise generated by the nearest road segment. The estimated hourly noise levels were validated against the traffic noise monitoring sites in Taipei City. The Pearson correlation coefficient between the estimated and measured hourly 24-h and nighttime noise levels were 0.70 and 0.54, respectively. The accuracy analysis showed that the hourly MAE and RMSE were 3.49 and 2.62 dBA for 24-h noise levels and 3.08 and 4.03 dBA for nighttime noise levels. Furthermore, the monthly average noise levels during the day, evening, and night (24 h, L_den_ indicator) and the average nighttime (23:00–6:00) exposure (L_night_ indicator) were calculated based on the noise prediction models for each road segment.

### Covariates

Covariates were selected for model adjustment based on a self-administered questionnaire administered prior to the final health checkup. This was divided into six categories: basic information, family health history, current health status, lifestyle, physical activity, sleep duration, and dietary habits. Basic information included age (20–39 years, 40–49 years, 50–59 years and > 60 years), sex, smoking status (never smoked, former smoker, current smoker), drinking status (never drank, former drinker, current drinker), education level (high school diploma or less, bachelor’s degree, and master's degree or above), and marital status (single, married/cohabitating, widowed, and divorced). Family health history of chronic diseases, which is a record of the relationships among family members along with their health histories, was categorized as yes or no. Biweekly physical activity was broadly classified into four categories (< 30 min, 30–60 min, 60–120 min, and > 120 min). The sleep durations were less than 6 h, 6–8 h and over 8 h. Eat-on time and regular amounts, at least 1 serving of rice or flour products, at least 1 serving of meat, at least 1 serving of vegetables, and at least 1 serving of fruits were categorized as yes or no, respectively.

### Statistical analysis

MetS and its five subcomponents were investigated separately as outcomes. Cox proportional hazards regression models with month as the underlying time scale were used to estimate hazard ratios (HRs) for the impact of PM_2.5_ and road traffic noise exposure on the risk of developing MetS or its subcomponents. Participants were censored if they did not have MetS at their final health checkup or if they were diagnosed with MetS at one of their previous health checkups.

Monthly PM_2.5_ and road traffic noise exposures were treated as continuous variables.

We first conducted single exposure analyses for PM_2.5_ and road traffic noise. Subsequently, a co-exposure analysis was conducted for both PM_2.5_ and road traffic noise. Finally, co-exposure analysis of both PM_2.5_ and nighttime noise was used to examine the harmful effects of road traffic noise during sleep. To investigate the interaction between air and noise pollution, we used the median as the cut-off value for each pollution exposure categories in the co-exposure models to divide valid participants into high and low-exposure groups. All Models were adjusted for basic information, family health history, lifestyle, physical activity, sleep duration, and dietary habits, which were considered as confounders, and investigated how these covariates modified the associations between the development of MetS or its subcomponents and environmental pollution exposure. Pearson’s chi-square tests were also performed to investigate the association between covariates and the frequency of incident MetS. The significance level was set at *p* < 0.05 for all statistical tests. All analyses were performed using the R software (version 4.0.4, R Development Core Team, https://www.r-project.org/). The maps created using the Free and Open Source QGIS by using Version 3.14.15 (https://www.qgis.org/en/site/).

### Ethical approval

Informed consent was obtained from all the participants. This study was approved by the Institutional Review Board on Biomedical Science Research, Academia Sinica (AS-IRB-BM-22008). Individually identifiable health data were removed and anonymized throughout the study. This study was performed in accordance with the Declaration of Helsinki and followed an approved protocol.

## Results

As shown in Table 1, the sex distribution was approximately balanced. Most participants were 20–39 years old (46.29%), married or cohabitating (65.62%), had a bachelor’s degree (62.11%), and a family health history of at least one chronic disease (56.17%). In terms of daily lifestyle, 13.87% and 13.91% of the participants were respectively current smokers and drinkers, and 74.23% had a sleep duration of 6–8 h. The biweekly physical activity for at least 30 min was 70.99%. In relation to dietary habits, the proportion of eating on schedule and in regular amounts was 72.60%. At least 1 serving of rice or flour products, meat, vegetables, and fruits were 94.90%, 96.13%, 94.95% and 73.33%, respectively. The results of the chi-square test, which measures the association between potential covariates and MetS, were also shown in Table 1. All covariates were significantly associated with incident MetS except for family health history, daily servings of vegetables and daily servings of fruits a day. Spearman’s correlations between pollution exposure (PM_2.5_ and noise) and ordinal covariates were shown in Table S1 of supplement material. No significant association was found between pollution exposure and any of the introduced ordinal covariates at a significance level of 0.05.

**Table 1:** General characteristics of the valid participants at their last health checkup.

Characteristics	Category (*N*, %)	MetS (*N* (%))	Non-MetS (*N* (%))	*P*-value
Sex				< 0.0001
	Male (5161, 47.91)	309 (5.99)	4852 (94.01)	
	Female (5612, 52.09)	108 (1.92)	5504 (98.08)	
Age				0.00016
	20–39 years (4987, 46.29)	152 (3.05)	4835 (96.95)	
	40–49 years (3512, 32.6)	149 (4.24)	3363 (95.76)	
	50–59 years (1624, 15.07)	80 (4.93)	1544 (95.07)	
	Above 60 years (650, 6.03)	36 (5.54)	614 (94.46)	
Education level				0.0067
	High school diploma or less (1310, 12.16)	71 (5.42)	1239 (94.58)	
	Bachelor degree (6691, 62.11)	250 (3.74)	6441 (96.26)	
	Master's degree or above (2772, 25.73)	96 (3.46)	2676 (96.54)	
Marital status				< 0.0001
	Single (3176, 29.48)	83 (2.61)	3093 (97.39)	
	Married/Cohabitating (7069, 65.62)	312 (4.41)	6757 (95.59)	
	Divorced (361, 3.35)	12 (3.32)	349 (96.68)	
	Widowed (167, 1.55)	10 (5.99)	157 (94.01)	
Family medical history				0.46375
	No (4722, 43.83)	175 (3.71)	4547 (96.29)	
	Yes (6051, 56.17)	242 (4)	5809 (96)	
Smoking status				0.46375
	Never smoker (8618, 80)	271 (3.14)	8347 (96.86)	
	Former smoker (661, 6.14)	46 (6.96)	615 (93.04)	
	Current smoker (1494, 13.87)	100 (6.69)	1394 (93.31)	< 0.0001
Drinking status				
	Never drinker (9170, 85.12)	322 (3.51)	8848 (96.49)	
	Formrt drinker (104, 0.97)	5 (4.81)	99 (95.19)	
	Current drinker (1499, 13.91)	90 (6)	1409 (94)	< 0.0001
Sleep duration				
	Less 6 h (2491, 23.12)	116 (4.66)	2375 (95.34)	
	6–8 h (7997, 74.23)	293 (3.66)	7704 (96.34)	
	Over 8 h (285, 2.65)	8 (2.81)	277 (97.19)	0.05176
Biweekly physical activity				
	Less 30 min (3125, 29.01)	140 (4.48)	2985 (95.52)	
	30–60 min (4584, 42.55)	172 (3.75)	4412 (96.25)	
	60–120 min (2338, 21.7)	69 (2.95)	2269 (97.05)	0.01221
	Over 120 min (726, 6.74)	36 (4.96)	690 (95.04)	
Eat on time and in regular amounts				
	No (2952, 27.4)	142 (4.81)	2810 (95.19)	
	Yes (7821, 72.6)	275 (3.52)	7546 (96.48)	
Daily servings of rice or flour products	Note: 1 serving is equivalent to 1 bowl of rice, two rice bowls of noodles, four slices of thin bread, 1 round bread, 2 sets of baked wheat bread and twisted cruller			0.01221
	None or less than 1 serving a day (549, 5.1)	20 (3.64)	529 (96.36)	
	At least 1 serving (10,224, 94.9)	397 (3.88)	9827 (96.12)	
Weekly servings of meat	Note: 1 serving is equivalent to 1 pork or beef steak, approximately palm size, or 1 chicken leg, 1 hamburger patty, or about 4 tablespoons of other lean meat			
	None or less than 1 serving a week (417, 3.87)	10 (2.4)	407 (97.6)	
	At least 1 serving (10,356, 96.13)	407 (3.93)	9949 (96.07)	0.00229
Daily servings of vegetables	Note: 1 serving is equivalent to 1 bowl of light or dark colored vegetables			
	None or less than ½ serving a day (598, 5.55)	25 (4.18)	573 (95.82)	
	At least ½ serving (10,175, 94.45)	392 (3.85)	9783 (96.15)	0.14411
Daily servings of fruits	Note: 1 serving is equivalent to half a medium sized apple, grapefruit or guava, 1 orange or kiwi, half a kilo of papaya or watermelon, 5 lychees , 12 grapes or longans			
	None or less than 1 serving a day (2873, 26.67)	104 (3.62)	2769 (96.38)	
	At least 1 serving (7900, 73.33)	313 (3.96)	7587 (96.04)	0.76794

In Table 2, the monthly PM_2.5_ concentrations ranged from 14.96 to 22.45 µg/m^3^ with mean and the median concentrations of 19.84 and 19.78 µg/m^3^, respectively. The estimated monthly average L_den_ ranged from 65.54 to 74.83 dBA with mean and median levels of 67.77 and 68.66 dBA across the cohort, respectively. Figures S1–S3 show the spatio-temporal variations in the year-to-year PM2.5, all-day noise (L_den_), and nighttime noise (L_night_) exposure levels of valid participants during the study period. There was a relatively small number of cases with impaired fasting glucose (*N* = 50). Due to the insufficient sample size for the Cox model, we could not assess the effect of environmental exposure on it in this study.

**Table 2:** Descriptive statistics of the study population’s monthly mean PM_2.5_ and monthly mean L_den_ noise exposures.

	Min	Q_1_	Median	Q_3_	Max	Mean	Standard deviation
Valid participates							
Monthly PM_2.5_ exposure (µg/m^3^)	14.96	19.32	19.78	20.37	22.45	19.84	0.78
Monthly L_den_ noise exposure (dBA)	65.54	67.26	67.77	69.35	74.83	68.66	2.06
Monthly L_night_ noise exposure (dBA)	59.99	61.78	62.23	63.61	68.52	63.01	1.78
Male							
Monthly PM_2.5_ exposure (µg/m^3^)	14.96	19.27	19.73	20.33	22.36	19.79	0.8
Monthly L_den_ noise exposure (dBA)	65.54	67.3	67.91	71.01	74.83	68.89	2.18
Monthly L_night_ noise exposure (dBA)	59.99	61.8	62.33	65.35	68.52	63.19	1.87
Female							
Monthly PM_2.5_ exposure (µg/m^3^)	15.07	19.35	19.83	20.42	22.45	19.88	0.76
Monthly L_den_ noise exposure (dBA)	65.73	67.24	67.67	68.52	74.77	68.46	1.92
Monthly L_night_ noise exposure (dBA)	60.34	61.76	62.18	62.99	68.29	62.85	1.68
With Mets							
Monthly PM_2.5_ exposure (µg/m^3^)	14.96	19.32	20.35	20.6	22.36	19.84	1.48
Monthly L_den_ noise exposure (dBA)	65.8	67.8	68.38	70.9	74.83	69.26	2.14
Monthly L_night_ noise exposure (dBA)	59.99	61.93	62.47	64.9	68.1	63.24	1.85
Without Mets							
Monthly PM_2.5_ exposure (µg/m^3^)	17	19.32	19.77	20.34	22.45	19.84	0.73
Monthly L_den_ noise exposure (dBA)	65.54	67.25	67.74	69.26	74.81	68.64	2.06
Monthly L_night_ noise exposure (dBA)	60.2	61.77	62.22	63.58	68.52	63	1.78

Table S2 shows the results for a single PM_2.5_ exposure models. A higher PM_2.5_ concentration was significantly and positively associated with and increased risk of MetS and its subcomponents. The adjusted HR = 1.88 per 1 µg/m^3^ monthly PM_2.5_ increase for developing MetS. The HRs of abdominal obesity, high blood pressure, high triglycerides, and low HDL cholesterol for a single PM_2.5_ exposure were 1.32, 1.37, 1.50 and 1.83 with per 1 µg/m^3^ increase in monthly PM_2.5_, respectively. In the single monthly average L_den_ exposure model (Table S3), an elevated monthly average L_den_ was significantly and positively associated with an increased risk of MetS and its components during the follow-up period. The adjusted HR of developing MetS was 1.10 per 1 dBA increase in monthly average L_den_. The HRs of abdominal obesity, high blood pressure, high triglycerides, and low HDL cholesterol were 1.05, 1.04, 1.07 and 1.09 per 1 dBA increase in the monthly average L_den_, respectively. Table S4 shows the risk of a single monthly average L_night_ exposure. The adjusted HR of incident MetS was 1.07 per 1 dBA increase in monthly average L_night_. The adjusted HRs of abdominal obesity, high blood pressure, high triglycerides, and low HDL cholesterol were 1.04, 1.04, 1.06 and 1.08 per 1 dBA increase in the monthly average L_night_.

Table 3 and Table S5 show the effects of PM_2.5_ and L_den_ co-exposure on MetS and its subcomponents. Both PM_2.5_ and L_den_ exposures were associated with significant risks of MetS, with adjusted HRs ranging from 1.91 for PM_2.5_ to 1.11 for L_den_ per unit higher exposure. Additionally, the adjusted HRs for PM_2.5_ of co-exposure to PM_2.5_ and L_den_ on the four subcomponents of MetS were 1.33 for abdominal obesity, 1.37 for high blood pressure, 1.51 for high triglycerides and 1.84 for low HDL cholesterol. The adjusted HRs for L_den_ on the four subcomponents of Mets were then 1.05 for abdominal obesity, 1.05 for high blood pressure, 1.07 for high triglycerides, and 1.09 for low HDL cholesterol.

**Table 3:** Effect estimates from the co-exposure models without interaction terms for PM_2.5_ (per 1 μg/m^3^ increase) and traffic noise (per 1 dBA increase).

Metabolic syndrome and components	Crude model	Adjusted model
	HR (95% CI)	HR (95% CI)
**Co-exposures to PM**_**2.5**_** and all-day noise**		
Metabolic syndrome		
Monthly PM_2.5_	2.14 (1.89, 2.41)	1.91 (1.69, 2.16)
Monthly L_den_	1.14 (1.09, 1.19)	1.11 (1.06, 1.16)
Abdominal obesity		
Monthly PM_2.5_	1.41 (1.33, 1.49)	1.33 (1.26, 1.41)
Monthly L_den_	1.06 (1.04, 1.09)	1.05 (1.03, 1.08)
High blood pressure		
Monthly PM_2.5_	1.51 (1.43, 1.60)	1.37 (1.30, 1.45)
Monthly L_den_	1.05 (1.03, 1.07)	1.05 (1.02, 1.07)
High triglycerides		
Monthly PM_2.5_	1.66 (1.57, 1.76)	1.51 (1.42, 1.60)
Monthly L_den_	1.09 (1.07, 1.12)	1.07 (1.05, 1.09)
Low HDL cholesterol		
Monthly PM_2.5_	1.90 (1.77, 2.05)	1.84 (1.71, 1.99)
Monthly L_den_	1.10 (1.06, 1.13)	1.09 (1.06, 1.12)
**Co-exposures to PM**_**2.5**_** and nighttime noise**		
Metabolic syndrome		
Monthly PM_2.5_	2.12 (1.88, 2.39)	1.90 (1.68, 2.14)
Monthly L_night_	1.10 (1.05, 1.16)	1.08 (1.02, 1.13)
Abdominal obesity		
Monthly PM_2.5_	1.40 (1.32, 1.49)	1.33 (1.25, 1.41)
Monthly L_night_	1.06 (1.03, 1.08)	1.04 (1.01, 1.07)
High blood pressure		
Monthly PM_2.5_	1.51 (1.43, 1.60)	1.37 (1.30, 1.45)
Monthly L_night_	1.04 (1.02, 1.07)	1.04 (1.01, 1.06)
High triglycerides		
Monthly PM_2.5_	1.65 (1.56, 1.75)	1.50 (1.42, 1.59)
Monthly L_den_	1.09 (1.06, 1.12)	1.06 (1.04, 1.09)
Low HDL cholesterol		
Monthly PM_2.5_	1.89 (1.76, 2.04)	1.84 (1.70, 1.98)
Monthly L_den_	1.09 (1.05, 1.13)	1.08 (1.04, 1.12)

The associations between co-exposure to PM_2.5_ and L_night_ and MetS and its subcomponents are presented in Table 3 and Table S6. The adjusted HRs of PM_2.5_ were 1.90 for MetS, 1.33 for abdominal obesity, 1.37 for high blood pressure, 1.50 for high triglycerides, and 1.84 for low HDL cholesterol. The adjusted HRs of L_night_ were 1.08 for MetS, 1.04 for abdominal obesity, 1.04 for high blood pressure, 1.06 for high triglycerides, and 1.08 for low HDL cholesterol.

Regarding the relationship between incident MetS or its subcomponents and individual characteristics in co-exposure models (Tables S5 and S6), female sex was found to have a positive association with the incidence of low HDL cholesterol, and a protective effect against increasing risks of MetS and other subcomponents. Older age groups were at an increased risk of developing high blood pressure. Higher education levels were significantly and negatively associated with MetS and its subcomponents. The married and cohabitating groups had a significant positive association with abdominal obesity and high triglyceride levels, while the widowed group had a positive association with MetS, abdominal obesity, and high triglycerides. Current smokers in this study were found to have an increased risk of developing MetS and its subcomponents, except for high blood pressure. Both biweekly physical activity and eating on time and in regular amounts showed protective effects against an increased risk of MetS. Family health history and smoking status were found to have an increased risk of developing high blood pressure. Among the four dietary patterns, “at least 1 serving of rice or flour products a day” showed protective effects against an increased risk of MetS, and “at least 1 serving of meat a week” was found to have an increased risk of developing abdominal obesity.

**Figure 2:**
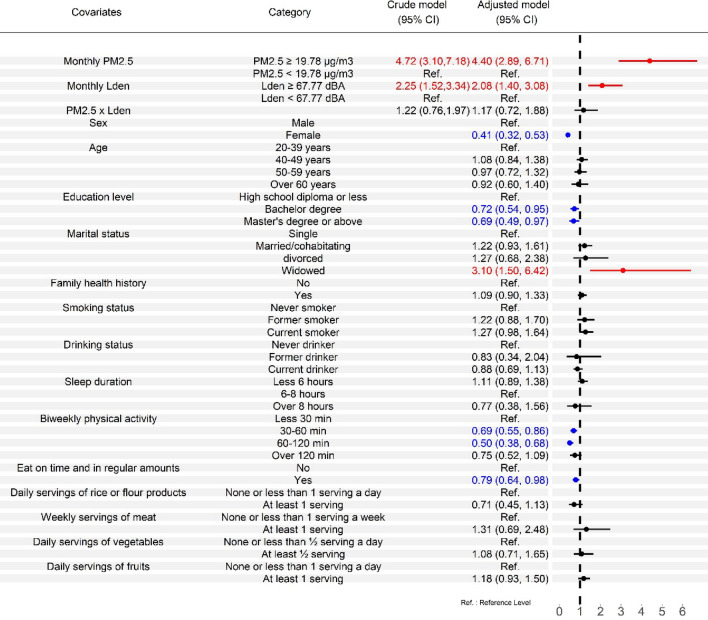
Effect estimates from the co-exposure models with interaction terms for PM_2.5_ and all-day traffic noise. Effect estimates are evaluated by hazard ratios. The crude model examines how PM_2.5_, all-day traffic noise, and the interaction term affect MetS and its subcomponents and ignores potential covariates. The adjusted model incorporates other potential covariates. The forest plot is used to visualize effect estimates and display the observed effects and confidence intervals.

**Figure 3:**
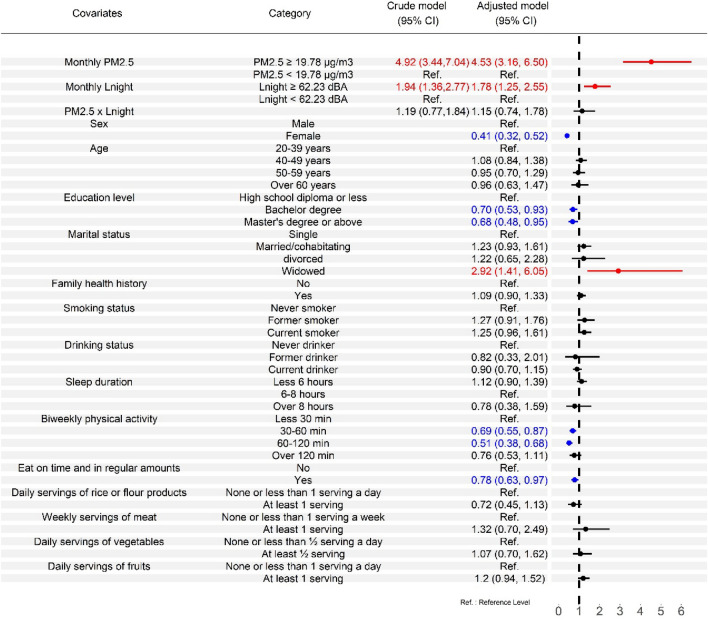
Effect estimates from the co-exposure models with interaction terms for PM_2.5_ and nighttime traffic noise. Effect estimates are evaluated by hazard ratios. The crude model examines how PM_2.5_, nighttime traffic noise, and the interaction term affect MetS and its subcomponents and ignores potential covariates. The adjusted model incorporates other potential covariates. The forest plot is used to visualize effect estimates and display the observed effects and confidence intervals.

Figure 2 reveals effect estimates from the co-exposure models with interaction terms for different exposure groups of PM_2.5_ and L_den_. Compared with the low PM_2.5_ exposure groups, the high exposure groups had significant higher adjusted HRs, ranging from 2.60 to 4.40, on MetS and its subcomponents after adjustment for potential confounders. The high L_den_ exposure groups also had significant higher adjusted HRs on MetS and its subcomponents (ranging from 1.58 to 2.22) compared with the reference groups. Figure 3 shows effect estimates from the co-exposure models with interaction terms for different exposure groups of PM_2.5_ and L_night_. Both high PM_2.5_ exposure groups and high L_night_ exposure groups had significant higher adjusted HRs on MetS and its subcomponents (ranging from 2.61 to 4.53 and 1.63 to 1.94, respectively) compared with their reference groups. Similar results were founded in Tables S9, S10, S13 and S14, except that high L_night_ exposure group of male participants was not statistically significant on Mets. However, the interactions of pollution exposures on MetS and its subcomponents were not statistically significant in all co-exposure models with interaction terms.

Table S7 demonstrates the effect estimates on MetS and its subcomponents from the co-exposure models for PM_2.5_ and L_den_ of valid male participants. Both PM_2.5_ and L_den_ exposures were associated with significant risks of MetS and its four subcomponents, with adjusted HRs ranging from 1.22 to 1.89 for PM_2.5_ and 1.03 to 1.08 for L_den_. Table S8 shows the effect estimates on MetS and its subcomponents from the co-exposure models for PM_2.5_ and Lnight of valid male participants. PM_2.5_ exposures were also associated with significant risks of MetS and its four subcomponents, with adjusted HRs ranging from 1.22 to 1.88. However, Lnight exposures were significant only for high triglycerides (adjusted HR = 1.06) and low HDL cholesterol (adjusted HR = 1.06). Table S11 shows the effect estimates on MetS and its subcomponents from the co-exposure models for PM_2.5_ and L_den_ of valid female participants. Both PM_2.5_ and L_den_ exposures were associated with significant risks of MetS and its four subcomponents, with adjusted HRs ranging from 1.49 to 2.14 for PM_2.5_ and 1.08 to 1.18 for L_den_. Table S12 shows the effect estimates on MetS and its subcomponents from the co-exposure models for PM_2.5_ and L_night_ of valid female participants. Both PM_2.5_ and L_night_ exposures were also associated with significant risks of MetS and its four subcomponents, with adjusted HRs ranging from 1.49 to 2.14 for PM_2.5_ and 1.06 to 1.14 for L_den_.

## Discussion

This study took advantage of finer air pollution and noise exposure data, along with repeated measurements from a health checkup database, to elucidate the incident risk of MetS and its subcomponents. Previous studies have focused on the links between environmental risk factors and the prevalence of MetS or its subcomponents in susceptible or specific races and ethnic groups^5,6,37,38^. The participants included in this study had a better health status at baseline, which is a good indicator to evaluate their progression towards MetS. The geocode addresses and fine timescales used for exposure assessment accurately reflect variations in local environmental exposures. Therefore, this study provides direct evidence of the adverse effects of air pollution and noise on incident MetS and its subcomponents in the general population. In addition, we adjusted for potential individual covariates. We found significant positive associations between PM_2.5_, noise (L_den_ and L_night_) exposure, and incident MetS in all the single and co-exposure models. Moreover, PM_2.5_ and noise (L_den_ and L_night_) exposures also significantly affected the incidence of MetS subcomponents during follow-up.

In this study, we find significantly effects of co-exposure to noise and PM_2.5_. on Mets and its subcomponents. The outcome was consistent with the finding in a previous study that evaluated associations between co-exposure groups and the prevalence of cardiovascular disease^34^. Eze et al.^39^ found that the combined impact of noise and air pollution on metabolic outcomes. Klompmaker et al.^40^ evaluated the combined impact of air and noise pollution on myocardial infarction. In addition, we did not find significantly interaction effects of co-exposure to noise and air pollution in the present study. Gan et al.^41^ found that there was not any positive interaction between noise and black carbon on risk of coronary heart disease mortality when they were assessed on either additive or multiplicative scales. Roswall et al.^42^ evaluated the combined effects of 10-year mean exposure to noise and NO_2_ in relation to risk of myocardial infarction. Results showed the highest HRs for both outcomes were among those with a combination of either high or medium noise exposure and high NO_2_ exposure. Thacher et al.^30^ evaluated associations between combined residential exposure to RTN and PM_2.5_ and cardiovascular disease (CVD) mortality risk (10-year mean exposure). In interaction analysis, results suggested that high exposure to both noise and PM_2.5_ resulted in the highest risk for CVD mortality.

Nighttime noise, especially that caused by road traffic, is considered a significant cause of sleep disturbances. Poor sleep causes measurable endocrine and metabolic disruptions and is associated with several negative cardiometabolic, psychiatric, and social outcomes^43–45^. Thus, we further assessed the effect of noise exposure during the nighttime (L_night_) on MetS and its subcomponents using co-exposure models. The risk of exposure to nighttime noise on MetS was slightly reduced compared to the risk of all-day noise exposure by adjusting for PM_2.5_ and individual covariates in co-exposure models. L_den_ added a constant penalty of 10 dBA for nighttime noise to account for the higher impact of nighttime noise than daytime noise. This might lead to a single L_night_ of exposure to MetS and its components having similar risks to a single L_den_ noise exposure. There may be two possible reasons why L_den_ had a greater impact on metabolic syndrome than did L_night_ in this study. First, there is a lot less traffic on the road at night than that during the day. Second, L_den_ is estimated based on noise levels over a whole day (24 h), with a penalty of 5 dB for the evening hours (19:00–23:00) and of 10 dB for the nighttime hours (23:00–06:00).

Evidence from epidemiological studies suggests that air pollution and noise trigger physiological alterations during the development of MetS^5,46^. Most studies have used annual mean exposure to environmental risk factors to assess associations with incident MetS or its subcomponents; however, a fine-scale temporal resolution may enhance our understanding of the impact of environmental risk factors. This study used a fine temporal resolution (monthly) to better understand the relationship among environmental factors, MetS, and its subcomponents.

Empirical and epidemiological evidence supports the concept that air pollution and noise have different underlying pathways that induce metabolic disorders. Plausible biological mechanisms for the effects of air pollution, especially PM_2.5_, on metabolic dysfunction include the induction of oxidative stress and inflammation in the lungs and migration through the lung epithelium into the bloodstream, thus contributing to adverse cardiometabolic outcomes^5,47,48^. Exposure to noise may induce stress and result in reduced sleep quality, which in turn can adversely affect heart rate, blood pressure, and changes in the metabolic system^44,49–51^. Thus, the mechanisms linking noise exposure and MetS risk may involve the stress response and may further result in metabolic dysfunction. These pathways may lead to systemic and vascular inflammation, increasing the risk of dyslipidemia, hypertension, thrombosis, insulin resistance, and consequently, metabolic alterations. However, due to insufficient information, it was not possible to characterize the effects of air pollution and noise on impaired fasting glucose. Our findings, which report significant effects of air and noise pollution on incident MetS and its subcomponents, were consistent with those reported in the literature.

Our study utilized an extensive selection process. By excluding all participants with incident MetS, the selected participants might have had a better health status than the overall population. On average, participants were younger and had a lower prevalence of MetS risk factors. This study had several limitations. (1) Covariate data were collected using a self-administered health questionnaire. This may have caused misclassification. Although data cleaning should have minimized this problem, a bias may still exist. (2) While exposure was assigned to the geocoded addresses of participants, we did not measure the amount of time the participants spent at home. Therefore, we do not know the extent to which the attributed values are consistent with the actual exposure of the subjects. (3) Both PM_2.5_ and noise exposures in outdoor environments were estimated based on the geocoded addresses of the participants. However, exposure to outdoor pollution may be more serious than that to indoor pollution. (4) Although we included several sets of covariates in the modeling analysis, variations in psychological stress should be considered in further studies. (5) In this study, we excluded participants who were on medication for hypertension, diabetes, or high blood lipids. Because no question on the duration of the medication was included in the questionnaire, we have no relevant information about it.

## Conclusion

Air and noise pollution are regarded as inevitable byproducts of urbanization. With rapid urbanization, the degree to which people are exposed to these two environmental pollutants is likely to increase. This study provides evidence based on the health checkup database that individual PM_2.5_ and noise, and their co-exposure may elevate the risk of MetS and its subcomponents. Therefore, prevention approaches should not only target exhaust emissions but also traffic noise. Stricter emission controls and changes in land-use and transportation programs that encourage public transit, car-sharing, and electric vehicles and generally reduce traffic may provide an effective avenue for reducing the incidence of MetS and generating large public health benefits.

### Supplementary Information

Supplementary Information.

## Data Availability

The data that support the findings of this study are available from the MJ Health Research Foundation, Taiwan. However, restrictions apply to the availability of these data, which are under approval for the current study and thus, are not publicly available. Data are however available from the corresponding authors upon reasonable request and with permission of MJ Health Research Foundation, Taiwan.

## Abbreviations

Mets: Metabolic syndrome
L_den_: Day evening night sound level
L_night_: Nighttime sound level
HRs: Hazard ratios
CVD: Cardiovascular disease
HDL: High-density lipoprotein
APD: Aerosol optical depth
MAE: Mean absolute error
RMSE: Root mean square error
dBA: A-weighted decibel
