# Supplementary material for: The impact of co-exposure to air and noise pollution on the incidence of metabolic syndrome from a health checkup cohort
Source: Sci Rep. 2024 Apr 17;14:8841. doi: 10.1038/s41598-024-59576-5 (PMC11024131; doi:10.1038/s41598-024-59576-5)
Supplement: Supplementary file 1 — Supplementary Information. [file 41598_2024_59576_MOESM1_ESM.docx]

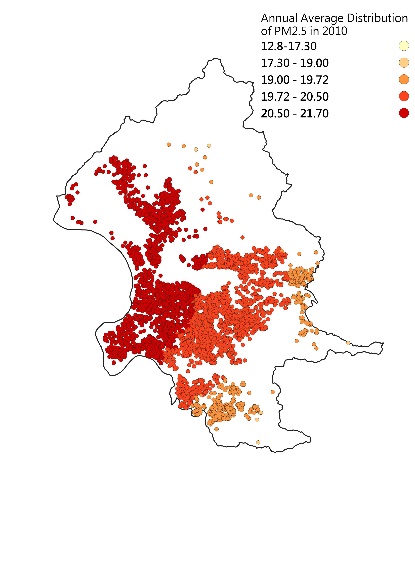

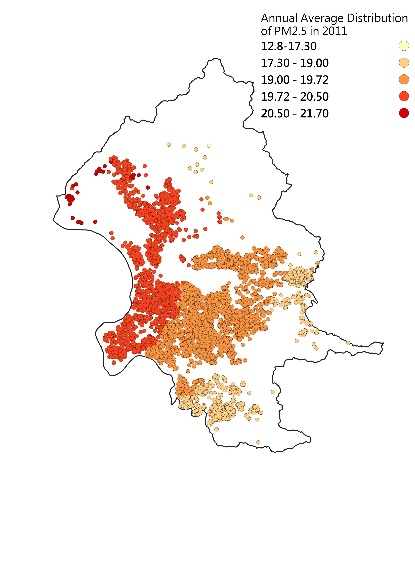

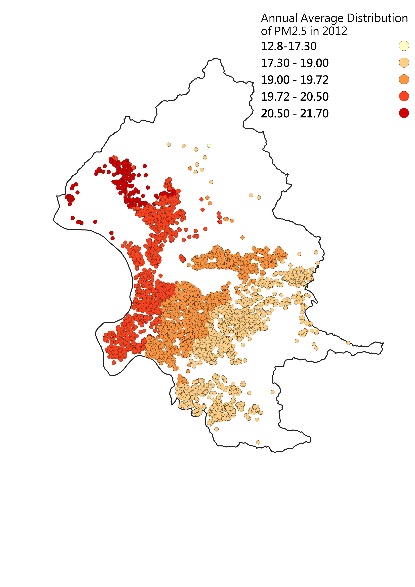


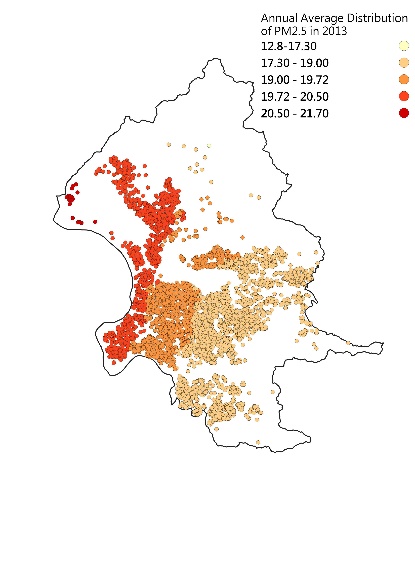

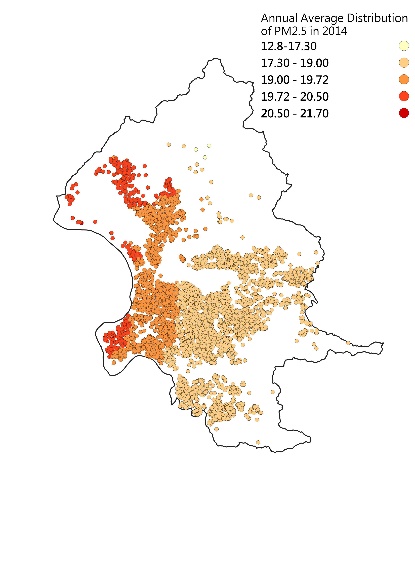

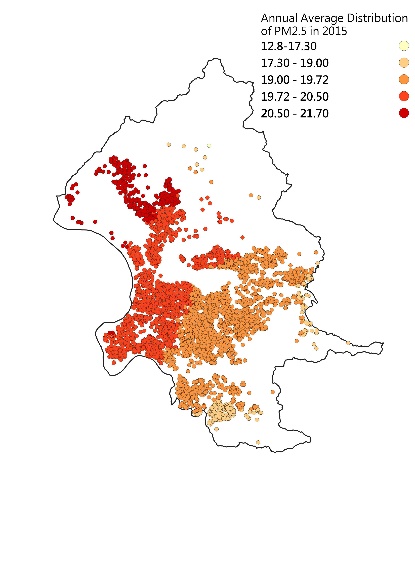


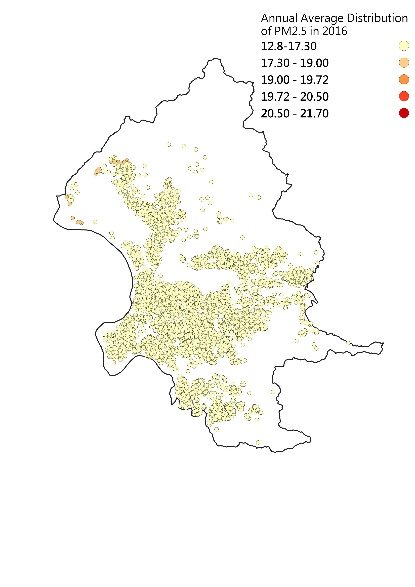


Figure S1 The spatio-temporal variations of the year-to-year PM_2.5_ exposure levels for valid participants during the study period. The map created using the Free and Open Source QGIS by using Version 3.14.15 (<https://www.qgis.org/en/site/>).


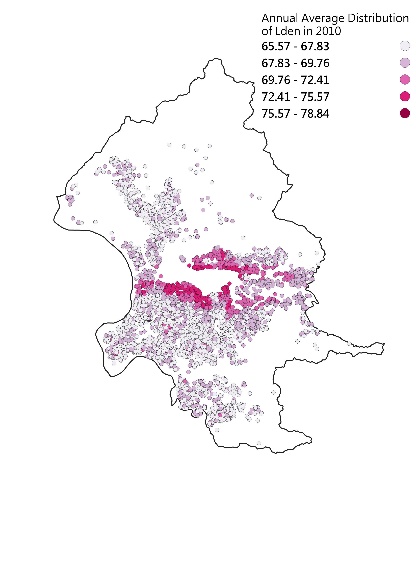

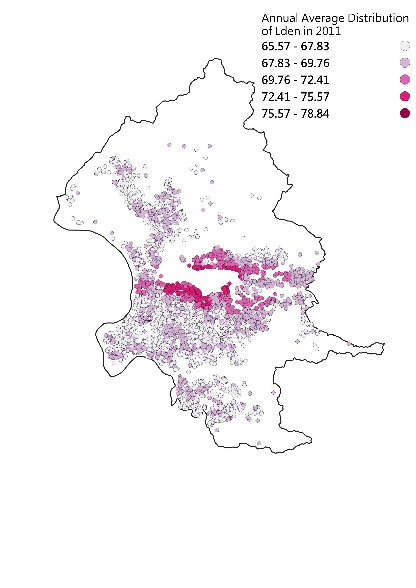

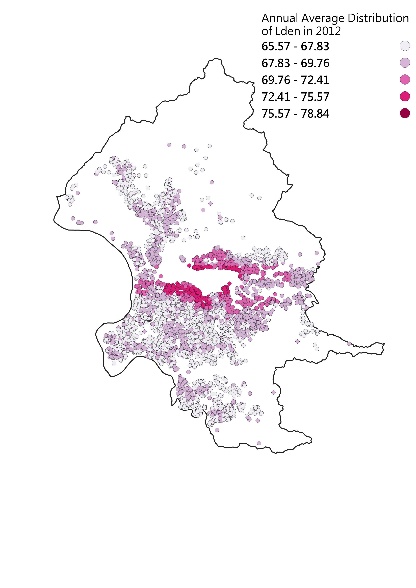


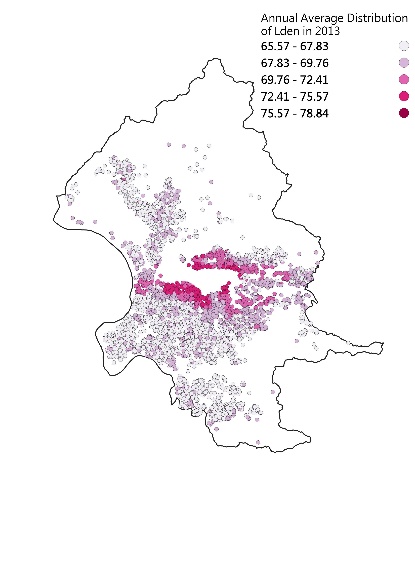

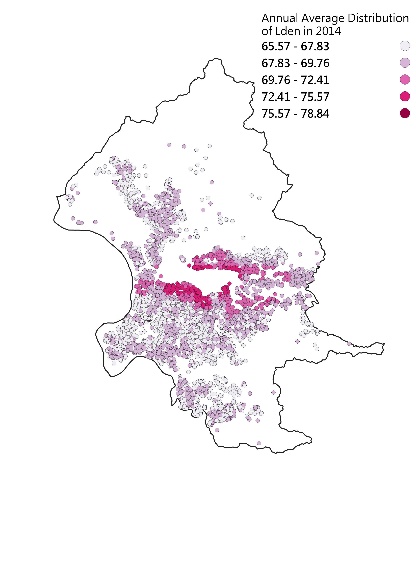

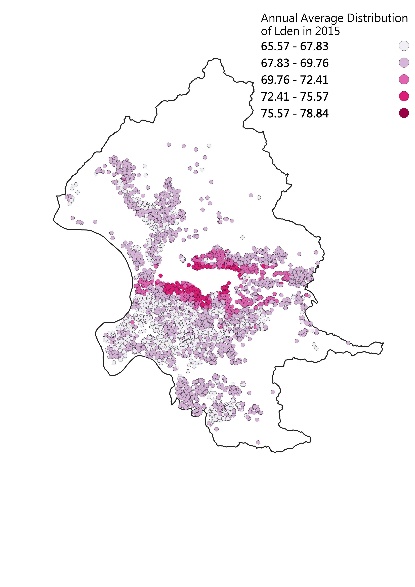


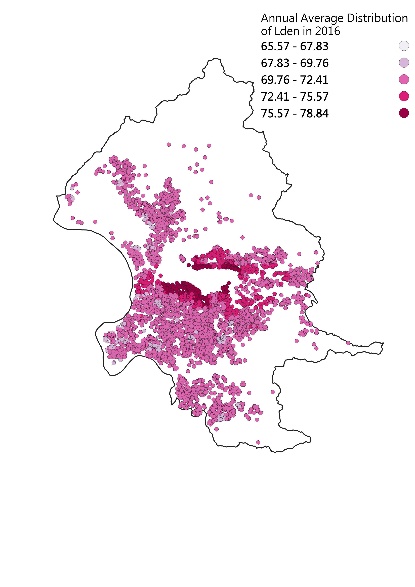


Figure S2 The spatio-temporal variations of the year-to-year day–evening–night noise levels (L_den_) for valid participants during the study period. The map created using the Free and Open Source QGIS by using Version 3.14.15 (<https://www.qgis.org/en/site/>).


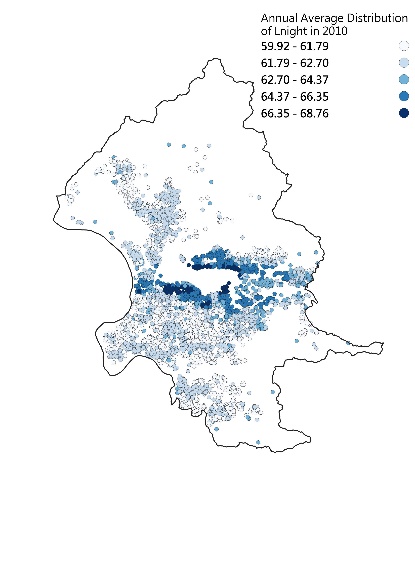

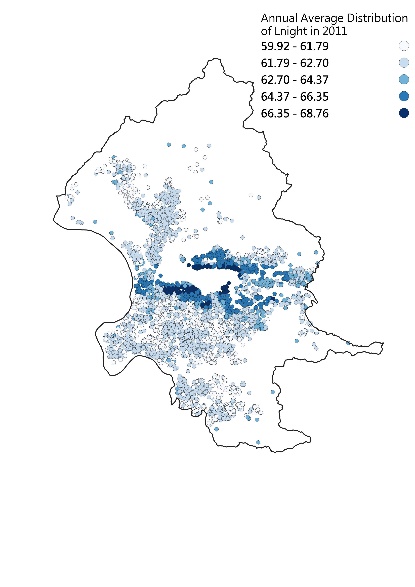

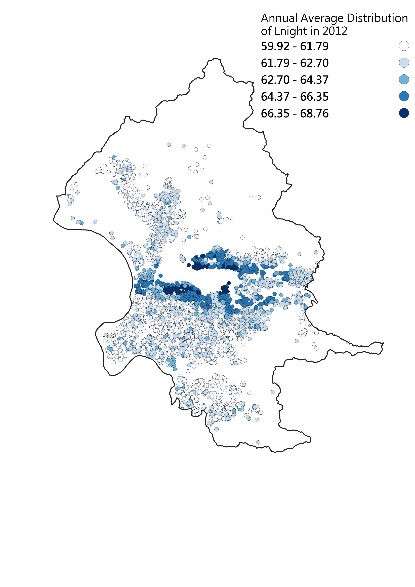


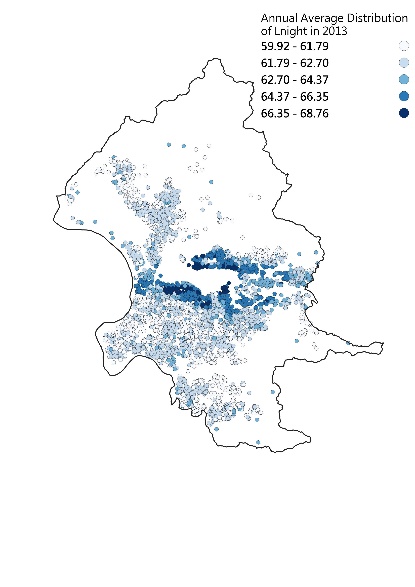

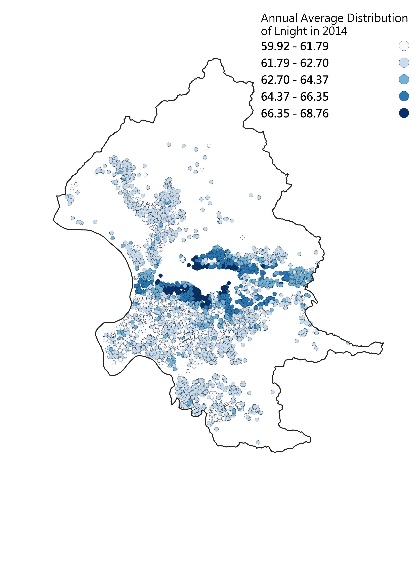

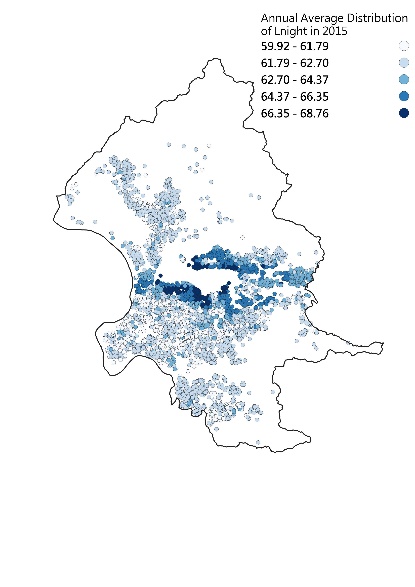


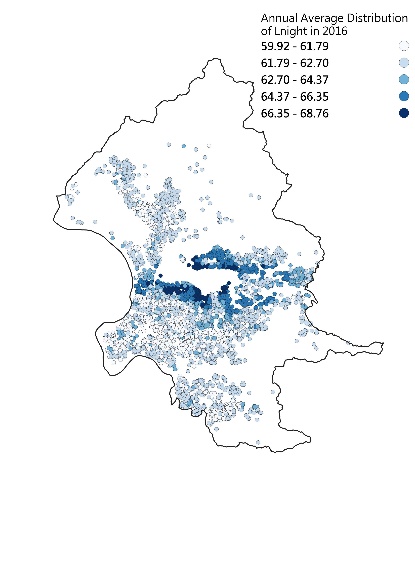


Figure S3 The spatio-temporal variations of the year-to-year nighttime noise levels (L_night_) for valid participants during the study period. The map created using the Free and Open Source QGIS by using Version 3.14.15 (<https://www.qgis.org/en/site/>).

Table S1 Spearman’s correlations between co-exposure (PM_2.5_ and traffic noise) and covariates.

|  | PM_2.5_ (μg/m^3^) | L_den_ (dBA) | Age group | Education level | Marital status | Family medical history | Smoking status | Drinking status | Biweekly physical activity | Sleep duration | Eat on time and in regular amounts |
| --- | --- | --- | --- | --- | --- | --- | --- | --- | --- | --- | --- |
| PM_2.5_ (μg/m^3^) | 1 | -0.09 | 0 | -0.11 | 0 | -0.03 | 0.02 | -0.01 | -0.04 | 0.01 | -0.04 |
| L_den_ (dBA) | -0.03 | 1 | -0.1 | 0.05 | -0.02 | 0 | 0.01 | 0.02 | 0.01 | -0.01 | -0.02 |
| Age | 0 | -0.08 | 1 | -0.23 | 0.45 | -0.06 | 0 | 0.01 | 0.08 | 0 | 0.14 |
| Education level | -0.11 | 0.06 | -0.23 | 1 | -0.11 | 0.07 | -0.1 | -0.03 | 0.03 | -0.07 | 0.08 |
| Marital status | 0 | -0.01 | 0.45 | -0.11 | 1 | -0.03 | 0.02 | 0.01 | 0.01 | -0.01 | 0.12 |
| Family medical history | -0.03 | 0 | -0.06 | 0.07 | -0.03 | 1 | -0.04 | -0.02 | -0.02 | 0.02 | -0.02 |
| Smoking status | 0.02 | 0.02 | 0 | -0.1 | 0.02 | -0.04 | 1 | 0.31 | 0.04 | 0.02 | -0.1 |
| Drinking status | -0.01 | 0.02 | 0.01 | -0.03 | 0.01 | -0.02 | 0.31 | 1 | 0.07 | 0.02 | -0.05 |
| Biweekly physical activity | -0.04 | 0.01 | 0.08 | 0.03 | 0.01 | -0.02 | 0.04 | 0.07 | 1 | -0.07 | 0.05 |
| Sleep duration | 0.01 | 0 | 0 | -0.07 | -0.01 | 0.02 | 0.02 | 0.02 | -0.07 | 1 | -0.11 |
| Eat on time and in regular amounts | -0.04 | -0.01 | 0.14 | 0.08 | 0.12 | -0.02 | -0.1 | -0.05 | 0.05 | -0.11 | 1 |

Table S2 Effect estimates from single exposure model for monthly PM_2.5_ exposure (per 1 μg/m^3^ increase)

| Covariates | Category | Metabolic syndrome | Abdominal obesity | High blood pressure | High triglycerides | Low HDL cholesterol |
| --- | --- | --- | --- | --- | --- | --- |
| Monthly PM_2.5_ |  | 1.88 (1.67, 2.12) | 1.32 (1.25, 1.4) | 1.37 (1.3, 1.45) | 1.5 (1.41, 1.58) | 1.83 (1.7, 1.97) |
| Sex | Male | Ref. | Ref. | Ref. | Ref. | Ref. |
|  | Female | 0.4 (0.31, 0.51) | 0.62 (0.56, 0.7) | 0.42 (0.37, 0.47) | 0.39 (0.35, 0.44) | 1.14 (0.99, 1.33) |
| Age | 20-39 years | Ref. | Ref. | Ref. | Ref. | Ref. |
|  | 40-49 years | 1.07 (0.83, 1.36) | 0.92 (0.81, 1.04) | 1.3 (1.15, 1.48) | 1.1 (0.98, 1.24) | 0.87 (0.75, 1.02) |
|  | 50-59 years | 0.95 (0.7, 1.28) | 0.81 (0.69, 0.95) | 1.56 (1.35, 1.81) | 1 (0.86, 1.16) | 0.66 (0.53, 0.82) |
|  | Over 60 years | 0.91 (0.6, 1.39) | 0.87 (0.7, 1.09) | 1.86 (1.54, 2.24) | 0.72 (0.57, 0.92) | 0.69 (0.51, 0.94) |
| Education level | High school diploma or less | Ref. | Ref. | Ref. | Ref. | Ref. |
|  | Bachelor degree | 0.67 (0.51, 0.89) | 0.83 (0.71, 0.97) | 0.79 (0.69, 0.9) | 0.83 (0.71, 0.96) | 0.78 (0.64, 0.96) |
|  | Master's degree or above | 0.6 (0.43, 0.85) | 0.7 (0.59, 0.84) | 0.74 (0.62, 0.86) | 0.84 (0.71, 1) | 0.79 (0.62, 1.01) |
| Marital status | Single | Ref. | Ref. | Ref. | Ref. | Ref. |
|  | Married/cohabitating | 1.25 (0.96, 1.64) | 1.33 (1.17, 1.52) | 0.94 (0.83, 1.08) | 1.31 (1.15, 1.49) | 1.12 (0.95, 1.31) |
|  | divorced | 1.14 (0.61, 2.14) | 1.23 (0.91, 1.68) | 0.9 (0.67, 1.2) | 1.59 (1.21, 2.11) | 1.07 (0.73, 1.57) |
|  | Widowed | 2.58 (1.25, 5.36) | 2.05 (1.4, 3) | 1.42 (1.04, 1.94) | 1.79 (1.14, 2.8) | 1.36 (0.77, 2.41) |
| Family health history | No | Ref. | Ref. | Ref. | Ref. | Ref. |
|  | Yes | 1.1 (0.91, 1.35) | 1.09 (0.99, 1.21) | 1.11 (1.01, 1.22) | 1.07 (0.97, 1.18) | 0.93 (0.81, 1.06) |
| Smoking status | Never smoker | Ref. | Ref. | Ref. | Ref. | Ref. |
|  | Former smoker | 1.3 (0.94, 1.81) | 1.25 (1.04, 1.5) | 1.03 (0.87, 1.22) | 1.07 (0.9, 1.27) | 1.09 (0.81, 1.47) |
|  | Current smoker | 1.4 (1.09, 1.81) | 1.17 (1.02, 1.35) | 0.92 (0.8, 1.05) | 1.23 (1.09, 1.4) | 1.44 (1.18, 1.75) |
| Drinking status | Never drinker | Ref. | Ref. | Ref. | Ref. | Ref. |
|  | Former drinker | 0.95 (0.39, 2.33) | 1.48 (0.97, 2.24) | 0.94 (0.64, 1.39) | 0.88 (0.56, 1.38) | 0.82 (0.39, 1.74) |
|  | Current drinker | 0.91 (0.71, 1.17) | 0.8 (0.7, 0.92) | 1.02 (0.9, 1.16) | 0.91 (0.8, 1.04) | 0.53 (0.42, 0.66) |
| Sleep duration | Less 6 hours | 1.05 (0.85, 1.31) | 1.08 (0.96, 1.2) | 1.03 (0.92, 1.15) | 0.94 (0.83, 1.05) | 0.89 (0.76, 1.03) |
|  | 6-8 hours | Ref. | Ref. | Ref. | Ref. | Ref. |
|  | Over 8 hours | 0.72 (0.36, 1.47) | 0.69 (0.47, 1.02) | 0.8 (0.58, 1.11) | 1.18 (0.89, 1.55) | 0.94 (0.63, 1.42) |
| Biweekly physical activity | Less 30 min | Ref. | Ref. | Ref. | Ref. | Ref. |
|  | 30-60 min | 0.67 (0.53, 0.84) | 0.81 (0.72, 0.91) | 0.92 (0.82, 1.04) | 0.79 (0.7, 0.89) | 0.81 (0.7, 0.94) |
|  | 60-120 min | 0.48 (0.36, 0.65) | 0.65 (0.56, 0.75) | 0.83 (0.72, 0.96) | 0.67 (0.58, 0.77) | 0.56 (0.46, 0.69) |
|  | Over 120 min | 0.69 (0.47, 1) | 0.92 (0.76, 1.12) | 0.92 (0.77, 1.11) | 0.78 (0.64, 0.94) | 0.63 (0.47, 0.85) |
| Eat on time and in regular amounts | No | Ref. | Ref. | Ref. | Ref. | Ref. |
|  | Yes | 0.74 (0.6, 0.92) | 0.73 (0.66, 0.82) | 0.93 (0.83, 1.04) | 0.94 (0.84, 1.05) | 0.88 (0.76, 1.02) |
| Daily servings of rice or flour products | None or less than 1 serving a day | Ref. | Ref. | Ref. | Ref. | Ref. |
|  | At least 1 serving | 0.71 (0.45, 1.12) | 0.94 (0.74, 1.19) | 1.02 (0.8, 1.3) | 1.03 (0.79, 1.35) | 1.19 (0.86, 1.65) |
| Weekly servings of meat | None or less than 1 serving a week | Ref. | Ref. | Ref. | Ref. | Ref. |
|  | At least 1 serving | 1.39 (0.73, 2.62) | 1.44 (1.05, 1.98) | 0.85 (0.67, 1.08) | 0.94 (0.71, 1.23) | 0.84 (0.62, 1.13) |
| Daily servings of vegetables | None or less than ½ serving a day | Ref. | Ref. | Ref. | Ref. | Ref. |
|  | At least ½ serving | 1.06 (0.7, 1.62) | 1.12 (0.89, 1.41) | 0.95 (0.77, 1.18) | 0.99 (0.8, 1.22) | 1.03 (0.77, 1.39) |
| Daily servings of fruits | None or less than 1 serving a day | Ref. | Ref. | Ref. | Ref. | Ref. |
|  | At least 1 serving | 1.23 (0.97, 1.56) | 0.92 (0.82, 1.03) | 1.08 (0.96, 1.22) | 1.1 (0.98, 1.23) | 1.16 (0.99, 1.36) |

*The effects are estimated by hazard ratios and their 95% CIs

Table S3 Effect estimates from singe exposure models for monthly all-day traffic noise exposure (per 1 dBA increase)

| Covariates | Category | Metabolic syndrome | Abdominal obesity | High blood pressure | High triglycerides | Low HDL cholesterol |
| --- | --- | --- | --- | --- | --- | --- |
| Monthly L_den_ |  | 1.1 (1.06, 1.15) | 1.05 (1.02, 1.07) | 1.04 (1.02, 1.07) | 1.07 (1.04, 1.09) | 1.09 (1.06, 1.12) |
| Sex | Male | Ref. | Ref. | Ref. | Ref. | Ref. |
|  | Female | 0.4 (0.31, 0.51) | 0.63 (0.56, 0.71) | 0.4 (0.36, 0.45) | 0.38 (0.34, 0.43) | 1.26 (1.08, 1.46) |
| Age | 20-39 years | Ref. | Ref. | Ref. | Ref. | Ref. |
|  | 40-49 years | 1.16 (0.91, 1.49) | 0.94 (0.83, 1.06) | 1.37 (1.21, 1.56) | 1.18 (1.05, 1.33) | 0.89 (0.76, 1.05) |
|  | 50-59 years | 1.1 (0.81, 1.49) | 0.84 (0.72, 0.99) | 1.71 (1.47, 1.98) | 1.1 (0.95, 1.28) | 0.68 (0.55, 0.85) |
|  | Over 60 years | 1.13 (0.74, 1.73) | 0.92 (0.74, 1.15) | 2.08 (1.73, 2.51) | 0.78 (0.61, 1) | 0.74 (0.54, 1.01) |
| Education level | High school diploma or less | Ref. | Ref. | Ref. | Ref. | Ref. |
|  | Bachelor degree | 0.6 (0.45, 0.79) | 0.8 (0.68, 0.93) | 0.73 (0.63, 0.83) | 0.76 (0.65, 0.88) | 0.69 (0.57, 0.85) |
|  | Master's degree or above | 0.5 (0.35, 0.7) | 0.65 (0.54, 0.78) | 0.66 (0.56, 0.77) | 0.73 (0.61, 0.87) | 0.63 (0.5, 0.8) |
| Marital status | Single | Ref. | Ref. | Ref. | Ref. | Ref. |
|  | Married/cohabitating | 1.26 (0.96, 1.65) | 1.35 (1.19, 1.54) | 0.94 (0.83, 1.07) | 1.31 (1.15, 1.5) | 1.13 (0.96, 1.33) |
|  | divorced | 1.06 (0.57, 1.99) | 1.21 (0.89, 1.65) | 0.9 (0.67, 1.21) | 1.57 (1.19, 2.07) | 1.1 (0.75, 1.62) |
|  | Widowed | 2.33 (1.12, 4.88) | 2.06 (1.4, 3.02) | 1.41 (1.03, 1.93) | 1.7 (1.08, 2.68) | 1.24 (0.7, 2.19) |
| Family health history | No | Ref. | Ref. | Ref. | Ref. | Ref. |
|  | Yes | 1.13 (0.93, 1.37) | 1.1 (0.99, 1.21) | 1.11 (1.01, 1.22) | 1.09 (0.99, 1.2) | 0.93 (0.82, 1.06) |
| Smoking status | Never smoker | Ref. | Ref. | Ref. | Ref. | Ref. |
|  | Former smoker | 1.3 (0.94, 1.81) | 1.28 (1.06, 1.53) | 0.98 (0.83, 1.16) | 1.08 (0.91, 1.29) | 1.05 (0.78, 1.41) |
|  | Current smoker | 1.48 (1.15, 1.91) | 1.21 (1.05, 1.4) | 0.93 (0.81, 1.06) | 1.33 (1.17, 1.51) | 1.55 (1.28, 1.88) |
| Drinking status | Never drinker | Ref. | Ref. | Ref. | Ref. | Ref. |
|  | Former drinker | 0.98 (0.4, 2.38) | 1.48 (0.98, 2.25) | 1 (0.68, 1.46) | 0.96 (0.61, 1.51) | 0.66 (0.31, 1.4) |
|  | Current drinker | 0.9 (0.7, 1.15) | 0.79 (0.68, 0.91) | 1.03 (0.91, 1.17) | 0.91 (0.8, 1.03) | 0.52 (0.42, 0.65) |
| Sleep duration | Less 6 hours | 1.04 (0.83, 1.29) | 1.07 (0.96, 1.2) | 1 (0.9, 1.12) | 0.93 (0.83, 1.04) | 0.85 (0.73, 0.99) |
|  | 6-8 hours | Ref. | Ref. | Ref. | Ref. | Ref. |
|  | Over 8 hours | 0.76 (0.37, 1.53) | 0.69 (0.47, 1.02) | 0.79 (0.57, 1.1) | 1.26 (0.96, 1.66) | 0.97 (0.64, 1.46) |
| Biweekly physical activity | Less 30 min | Ref. | Ref. | Ref. | Ref. | Ref. |
|  | 30-60 min | 0.63 (0.5, 0.79) | 0.78 (0.69, 0.88) | 0.91 (0.81, 1.02) | 0.76 (0.68, 0.86) | 0.77 (0.66, 0.9) |
|  | 60-120 min | 0.46 (0.35, 0.62) | 0.63 (0.54, 0.73) | 0.82 (0.71, 0.94) | 0.66 (0.57, 0.76) | 0.55 (0.45, 0.66) |
|  | Over 120 min | 0.62 (0.43, 0.91) | 0.87 (0.72, 1.06) | 0.89 (0.74, 1.07) | 0.74 (0.61, 0.89) | 0.62 (0.46, 0.83) |
| Eat on time and in regular amounts | No | Ref. | Ref. | Ref. | Ref. | Ref. |
|  | Yes | 0.72 (0.58, 0.89) | 0.71 (0.63, 0.79) | 0.92 (0.82, 1.02) | 0.91 (0.82, 1.02) | 0.85 (0.73, 0.99) |
| Daily servings of rice or flour products | None or less than 1 serving a day | Ref. | Ref. | Ref. | Ref. | Ref. |
|  | At least 1 serving | 0.69 (0.43, 1.08) | 0.91 (0.72, 1.16) | 0.98 (0.77, 1.25) | 1.03 (0.79, 1.35) | 1.19 (0.86, 1.65) |
| Weekly servings of meat | None or less than 1 serving a week | Ref. | Ref. | Ref. | Ref. | Ref. |
|  | At least 1 serving | 1.52 (0.8, 2.87) | 1.51 (1.1, 2.07) | 0.85 (0.67, 1.07) | 0.99 (0.75, 1.29) | 0.84 (0.62, 1.13) |
| Daily servings of vegetables | None or less than ½ serving a day | Ref. | Ref. | Ref. | Ref. | Ref. |
|  | At least ½ serving | 0.93 (0.61, 1.42) | 1.1 (0.88, 1.39) | 0.91 (0.73, 1.12) | 0.91 (0.74, 1.13) | 1.04 (0.78, 1.39) |
| Daily servings of fruits | None or less than 1 serving a day | Ref. | Ref. | Ref. | Ref. | Ref. |
|  | At least 1 serving | 1.2 (0.95, 1.53) | 0.92 (0.82, 1.03) | 1.08 (0.96, 1.21) | 1.09 (0.97, 1.23) | 1.13 (0.97, 1.33) |

*The effects are estimated by hazard ratios and their 95% CIs

Table S4 Effect estimates from singe exposure models for monthly average nighttime traffic noise exposure (per 1 dBA increase)

| Covariates | Category | Metabolic syndrome | Abdominal obesity | High blood pressure | High triglycerides | Low HDL cholesterol |
| --- | --- | --- | --- | --- | --- | --- |
| Monthly L_night_ |  | 1.07 (1.02, 1.13) | 1.04 (1.01, 1.07) | 1.04 (1.01, 1.06) | 1.06 (1.03, 1.09) | 1.08 (1.04, 1.12) |
| Sex | Male | Ref. | Ref. | Ref. | Ref. | Ref. |
|  | Female | 0.39 (0.31, 0.5) | 0.63 (0.56, 0.7) | 0.4 (0.35, 0.45) | 0.38 (0.34, 0.43) | 1.24 (1.07, 1.44) |
| Age | 20-39 years | Ref. | Ref. | Ref. | Ref. | Ref. |
|  | 40-49 years | 1.15 (0.9, 1.47) | 0.93 (0.82, 1.05) | 1.37 (1.2, 1.55) | 1.17 (1.04, 1.32) | 0.89 (0.76, 1.04) |
|  | 50-59 years | 1.07 (0.79, 1.44) | 0.83 (0.71, 0.98) | 1.7 (1.46, 1.97) | 1.09 (0.94, 1.27) | 0.67 (0.54, 0.84) |
|  | Over 60 years | 1.09 (0.71, 1.66) | 0.91 (0.73, 1.14) | 2.06 (1.71, 2.49) | 0.77 (0.6, 0.98) | 0.73 (0.54, 1) |
| Education level | High school diploma or less | Ref. | Ref. | Ref. | Ref. | Ref. |
|  | Bachelor degree | 0.61 (0.46, 0.8) | 0.8 (0.69, 0.94) | 0.73 (0.63, 0.83) | 0.76 (0.65, 0.88) | 0.7 (0.57, 0.85) |
|  | Master's degree or above | 0.51 (0.36, 0.72) | 0.65 (0.55, 0.79) | 0.66 (0.56, 0.77) | 0.74 (0.62, 0.88) | 0.64 (0.5, 0.81) |
| Marital status | Single | Ref. | Ref. | Ref. | Ref. | Ref. |
|  | Married/cohabitating | 1.26 (0.96, 1.65) | 1.35 (1.19, 1.54) | 0.94 (0.83, 1.07) | 1.31 (1.15, 1.5) | 1.13 (0.96, 1.33) |
|  | divorced | 1.08 (0.58, 2.02) | 1.22 (0.89, 1.66) | 0.9 (0.67, 1.21) | 1.57 (1.19, 2.08) | 1.1 (0.75, 1.63) |
|  | Widowed | 2.41 (1.15, 5.04) | 2.08 (1.42, 3.05) | 1.42 (1.04, 1.95) | 1.72 (1.1, 2.71) | 1.25 (0.71, 2.21) |
| Family health history | No | Ref. | Ref. | Ref. | Ref. | Ref. |
|  | Yes | 1.12 (0.92, 1.37) | 1.09 (0.99, 1.21) | 1.11 (1.01, 1.22) | 1.08 (0.98, 1.19) | 0.93 (0.82, 1.06) |
| Smoking status | Never smoker | Ref. | Ref. | Ref. | Ref. | Ref. |
|  | Former smoker | 1.32 (0.95, 1.84) | 1.28 (1.06, 1.54) | 0.98 (0.83, 1.17) | 1.09 (0.91, 1.3) | 1.05 (0.78, 1.42) |
|  | Current smoker | 1.49 (1.15, 1.93) | 1.21 (1.05, 1.4) | 0.92 (0.8, 1.06) | 1.33 (1.17, 1.51) | 1.55 (1.28, 1.88) |
| Drinking status | Never drinker | Ref. | Ref. | Ref. | Ref. | Ref. |
|  | Former drinker | 0.96 (0.4, 2.35) | 1.48 (0.97, 2.24) | 0.99 (0.68, 1.46) | 0.94 (0.6, 1.49) | 0.66 (0.31, 1.4) |
|  | Current drinker | 0.9 (0.7, 1.16) | 0.79 (0.68, 0.91) | 1.03 (0.91, 1.16) | 0.91 (0.8, 1.03) | 0.52 (0.42, 0.65) |
| Sleep duration | Less 6 hours | 1.04 (0.84, 1.3) | 1.07 (0.96, 1.2) | 1.01 (0.9, 1.12) | 0.93 (0.83, 1.04) | 0.85 (0.73, 0.99) |
|  | 6-8 hours | Ref. | Ref. | Ref. | Ref. | Ref. |
|  | Over 8 hours | 0.76 (0.38, 1.55) | 0.69 (0.47, 1.02) | 0.8 (0.58, 1.11) | 1.26 (0.96, 1.66) | 0.97 (0.64, 1.46) |
| Biweekly physical activity | Less 30 min | Ref. | Ref. | Ref. | Ref. | Ref. |
|  | 30-60 min | 0.62 (0.5, 0.78) | 0.78 (0.69, 0.88) | 0.91 (0.8, 1.02) | 0.76 (0.68, 0.86) | 0.77 (0.66, 0.89) |
|  | 60-120 min | 0.46 (0.34, 0.62) | 0.63 (0.54, 0.73) | 0.82 (0.71, 0.94) | 0.66 (0.57, 0.76) | 0.54 (0.45, 0.66) |
|  | Over 120 min | 0.62 (0.43, 0.91) | 0.88 (0.72, 1.06) | 0.9 (0.75, 1.08) | 0.74 (0.61, 0.89) | 0.62 (0.46, 0.83) |
| Eat on time and in regular amounts | No | Ref. | Ref. | Ref. | Ref. | Ref. |
|  | Yes | 0.72 (0.58, 0.89) | 0.71 (0.64, 0.79) | 0.91 (0.82, 1.02) | 0.91 (0.82, 1.02) | 0.85 (0.73, 0.99) |
| Daily servings of rice or flour products | None or less than 1 serving a day | Ref. | Ref. | Ref. | Ref. | Ref. |
|  | At least 1 serving | 0.69 (0.44, 1.09) | 0.91 (0.72, 1.16) | 0.98 (0.77, 1.25) | 1.03 (0.79, 1.35) | 1.19 (0.86, 1.65) |
| Weekly servings of meat | None or less than 1 serving a week | Ref. | Ref. | Ref. | Ref. | Ref. |
|  | At least 1 serving | 1.49 (0.79, 2.81) | 1.5 (1.09, 2.06) | 0.84 (0.67, 1.07) | 0.98 (0.74, 1.28) | 0.83 (0.62, 1.12) |
| Daily servings of vegetables | None or less than ½ serving a day | Ref. | Ref. | Ref. | Ref. | Ref. |
|  | At least ½ serving | 0.93 (0.61, 1.42) | 1.11 (0.88, 1.4) | 0.92 (0.74, 1.14) | 0.92 (0.74, 1.14) | 1.04 (0.78, 1.4) |
| Daily servings of fruits | None or less than 1 serving a day | Ref. | Ref. | Ref. | Ref. | Ref. |
|  | At least 1 serving | 1.21 (0.96, 1.54) | 0.92 (0.82, 1.03) | 1.08 (0.96, 1.21) | 1.1 (0.98, 1.23) | 1.14 (0.97, 1.33) |

*The effects are estimated by hazard ratios and their 95% CIs

Table S5 Effect estimates from the co-exposure models for monthly PM_2.5_ exposure (per 1 μg/m^3^ increase) and all-day traffic noise exposure (per 1 dBA increase)

| Covariates | Category | Metabolic syndrome | Abdominal obesity | High blood pressure | High triglycerides | Low HDL cholesterol |
| --- | --- | --- | --- | --- | --- | --- |
| Monthly PM_2.5_ |  | 1.91 (1.69, 2.16) | 1.33 (1.26, 1.41) | 1.37 (1.3, 1.45) | 1.51 (1.42, 1.6) | 1.84 (1.71, 1.99) |
| Monthly L_den_ |  | 1.11 (1.06, 1.16) | 1.05 (1.03, 1.08) | 1.05 (1.02, 1.07) | 1.07 (1.05, 1.09) | 1.09 (1.06, 1.12) |
| Sex | Male | Ref. | Ref. | Ref. | Ref. | Ref. |
|  | Female | 0.41 (0.32, 0.53) | 0.64 (0.57, 0.71) | 0.42 (0.38, 0.47) | 0.4 (0.36, 0.46) | 1.18 (1.02, 1.37) |
| Age | 20-39 years | Ref. | Ref. | Ref. | Ref. | Ref. |
|  | 40-49 years | 1.11 (0.87, 1.42) | 0.93 (0.82, 1.05) | 1.32 (1.16, 1.5) | 1.14 (1.01, 1.28) | 0.89 (0.76, 1.04) |
|  | 50-59 years | 1.02 (0.75, 1.38) | 0.83 (0.71, 0.98) | 1.6 (1.38, 1.86) | 1.05 (0.91, 1.23) | 0.7 (0.56, 0.87) |
|  | Over 60 years | 0.99 (0.65, 1.52) | 0.91 (0.73, 1.14) | 1.94 (1.61, 2.34) | 0.78 (0.61, 0.99) | 0.74 (0.54, 1) |
| Education level | High school diploma or less | Ref. | Ref. | Ref. | Ref. | Ref. |
|  | Bachelor degree | 0.66 (0.49, 0.87) | 0.83 (0.71, 0.96) | 0.78 (0.68, 0.9) | 0.81 (0.7, 0.94) | 0.78 (0.64, 0.95) |
|  | Master's degree or above | 0.58 (0.42, 0.82) | 0.69 (0.58, 0.83) | 0.73 (0.62, 0.86) | 0.82 (0.69, 0.98) | 0.78 (0.61, 1) |
| Marital status | Single | Ref. | Ref. | Ref. | Ref. | Ref. |
|  | Married/cohabitating | 1.25 (0.95, 1.64) | 1.33 (1.17, 1.52) | 0.95 (0.83, 1.08) | 1.3 (1.14, 1.48) | 1.12 (0.95, 1.32) |
|  | divorced | 1.1 (0.59, 2.06) | 1.21 (0.89, 1.65) | 0.89 (0.66, 1.19) | 1.57 (1.19, 2.07) | 1.04 (0.7, 1.52) |
|  | Widowed | 2.4 (1.15, 4.99) | 2 (1.36, 2.93) | 1.39 (1.02, 1.9) | 1.72 (1.09, 2.7) | 1.3 (0.74, 2.3) |
| Family health history | No | Ref. | Ref. | Ref. | Ref. | Ref. |
|  | Yes | 1.12 (0.92, 1.37) | 1.1 (1, 1.22) | 1.11 (1.01, 1.22) | 1.08 (0.98, 1.19) | 0.94 (0.82, 1.07) |
| Smoking status | Never smoker | Ref. | Ref. | Ref. | Ref. | Ref. |
|  | Former smoker | 1.25 (0.9, 1.74) | 1.25 (1.04, 1.5) | 1.02 (0.86, 1.21) | 1.04 (0.87, 1.25) | 1.07 (0.79, 1.44) |
|  | Current smoker | 1.37 (1.06, 1.78) | 1.18 (1.02, 1.36) | 0.92 (0.8, 1.05) | 1.22 (1.08, 1.39) | 1.42 (1.17, 1.72) |
| Drinking status | Never drinker | Ref. | Ref. | Ref. | Ref. | Ref. |
|  | Former drinker | 0.96 (0.39, 2.35) | 1.48 (0.97, 2.24) | 0.94 (0.64, 1.38) | 0.92 (0.58, 1.45) | 0.8 (0.38, 1.7) |
|  | Current drinker | 0.9 (0.7, 1.16) | 0.8 (0.69, 0.92) | 1.03 (0.91, 1.16) | 0.91 (0.8, 1.04) | 0.53 (0.42, 0.67) |
| Sleep duration | Less 6 hours | 1.04 (0.84, 1.3) | 1.07 (0.96, 1.2) | 1.02 (0.91, 1.14) | 0.93 (0.83, 1.05) | 0.88 (0.75, 1.02) |
|  | 6-8 hours | Ref. | Ref. | Ref. | Ref. | Ref. |
|  | Over 8 hours | 0.75 (0.37, 1.52) | 0.69 (0.47, 1.01) | 0.8 (0.58, 1.1) | 1.19 (0.91, 1.57) | 0.97 (0.64, 1.46) |
| Biweekly physical activity | Less 30 min | Ref. | Ref. | Ref. | Ref. | Ref. |
|  | 30-60 min | 0.68 (0.54, 0.85) | 0.81 (0.72, 0.91) | 0.92 (0.82, 1.04) | 0.79 (0.71, 0.89) | 0.82 (0.7, 0.95) |
|  | 60-120 min | 0.49 (0.37, 0.66) | 0.66 (0.57, 0.76) | 0.83 (0.73, 0.96) | 0.68 (0.59, 0.78) | 0.58 (0.48, 0.71) |
|  | Over 120 min | 0.68 (0.47, 0.98) | 0.92 (0.76, 1.12) | 0.91 (0.75, 1.09) | 0.76 (0.63, 0.92) | 0.63 (0.47, 0.85) |
| Eat on time and in regular amounts | No | Ref. | Ref. | Ref. | Ref. | Ref. |
|  | Yes | 0.74 (0.6, 0.92) | 0.73 (0.65, 0.82) | 0.93 (0.83, 1.04) | 0.94 (0.84, 1.05) | 0.88 (0.76, 1.02) |
| Daily servings of rice or flour products | None or less than 1 serving a day | Ref. | Ref. | Ref. | Ref. | Ref. |
|  | At least 1 serving | 0.7 (0.44, 1.1) | 0.93 (0.73, 1.19) | 1.02 (0.8, 1.29) | 1.02 (0.78, 1.34) | 1.18 (0.85, 1.63) |
| Weekly servings of meat | None or less than 1 serving a week | Ref. | Ref. | Ref. | Ref. | Ref. |
|  | At least 1 serving | 1.44 (0.76, 2.72) | 1.46 (1.07, 2.01) | 0.87 (0.69, 1.1) | 0.96 (0.73, 1.26) | 0.83 (0.62, 1.12) |
| Daily servings of vegetables | None or less than ½ serving a day | Ref. | Ref. | Ref. | Ref. | Ref. |
|  | At least ½ serving | 1.04 (0.69, 1.59) | 1.11 (0.88, 1.4) | 0.94 (0.76, 1.16) | 0.97 (0.78, 1.2) | 1.02 (0.76, 1.37) |
| Daily servings of fruits | None or less than 1 serving a day | Ref. | Ref. | Ref. | Ref. | Ref. |
|  | At least 1 serving | 1.21 (0.95, 1.53) | 0.92 (0.82, 1.03) | 1.08 (0.96, 1.21) | 1.09 (0.97, 1.23) | 1.15 (0.98, 1.35) |

*The effects are estimated by hazard ratios and their 95% CIs

Table S6 Effect estimates from the co-exposure models for monthly PM_2.5_ exposure (per 1 μg/m^3^ increase) and nighttime traffic noise exposure (per 1 dBA increase)

| Covariates | Category | Metabolic syndrome | Abdominal obesity | High blood pressure | High triglycerides | Low HDL cholesterol |
| --- | --- | --- | --- | --- | --- | --- |
| Monthly PM_2.5_ |  | 1.9 (1.68, 2.14) | 1.33 (1.25, 1.41) | 1.37 (1.3, 1.45) | 1.5 (1.42, 1.59) | 1.84 (1.7, 1.98) |
| Monthly L_night_ |  | 1.08 (1.02, 1.13) | 1.04 (1.01, 1.07) | 1.04 (1.01, 1.06) | 1.06 (1.04, 1.09) | 1.08 (1.04, 1.12) |
| Sex | Male | Ref. | Ref. | Ref. | Ref. | Ref. |
|  | Female | 0.41 (0.32, 0.52) | 0.63 (0.56, 0.71) | 0.42 (0.37, 0.47) | 0.4 (0.36, 0.45) | 1.17 (1.01, 1.36) |
| Age | 20-39 years | Ref. | Ref. | Ref. | Ref. | Ref. |
|  | 40-49 years | 1.09 (0.85, 1.4) | 0.93 (0.82, 1.05) | 1.32 (1.16, 1.49) | 1.13 (1, 1.27) | 0.89 (0.76, 1.04) |
|  | 50-59 years | 0.99 (0.73, 1.34) | 0.83 (0.7, 0.97) | 1.59 (1.37, 1.85) | 1.04 (0.9, 1.21) | 0.69 (0.56, 0.86) |
|  | Over 60 years | 0.96 (0.63, 1.47) | 0.9 (0.72, 1.12) | 1.92 (1.59, 2.31) | 0.76 (0.6, 0.98) | 0.73 (0.53, 1) |
| Education level | High school diploma or less | Ref. | Ref. | Ref. | Ref. | Ref. |
|  | Bachelor degree | 0.66 (0.5, 0.88) | 0.83 (0.71, 0.97) | 0.79 (0.68, 0.9) | 0.81 (0.7, 0.95) | 0.78 (0.64, 0.95) |
|  | Master's degree or above | 0.6 (0.43, 0.84) | 0.7 (0.58, 0.84) | 0.73 (0.62, 0.86) | 0.83 (0.7, 0.99) | 0.79 (0.62, 1.01) |
| Marital status | Single | Ref. | Ref. | Ref. | Ref. | Ref. |
|  | Married/cohabitating | 1.25 (0.96, 1.64) | 1.33 (1.17, 1.52) | 0.95 (0.83, 1.08) | 1.3 (1.14, 1.48) | 1.12 (0.95, 1.32) |
|  | divorced | 1.12 (0.6, 2.1) | 1.22 (0.9, 1.67) | 0.89 (0.66, 1.19) | 1.58 (1.19, 2.08) | 1.05 (0.71, 1.54) |
|  | Widowed | 2.49 (1.2, 5.17) | 2.02 (1.38, 2.96) | 1.4 (1.02, 1.91) | 1.74 (1.11, 2.73) | 1.32 (0.74, 2.32) |
| Family health history | No | Ref. | Ref. | Ref. | Ref. | Ref. |
|  | Yes | 1.12 (0.92, 1.36) | 1.1 (1, 1.22) | 1.11 (1.01, 1.22) | 1.08 (0.98, 1.19) | 0.94 (0.82, 1.07) |
| Smoking status | Never smoker | Ref. | Ref. | Ref. | Ref. | Ref. |
|  | Former smoker | 1.27 (0.91, 1.77) | 1.25 (1.04, 1.5) | 1.02 (0.86, 1.21) | 1.05 (0.88, 1.25) | 1.07 (0.79, 1.44) |
|  | Current smoker | 1.38 (1.07, 1.79) | 1.18 (1.02, 1.35) | 0.91 (0.8, 1.05) | 1.22 (1.08, 1.39) | 1.42 (1.17, 1.73) |
| Drinking status | Never drinker | Ref. | Ref. | Ref. | Ref. | Ref. |
|  | Former drinker | 0.95 (0.39, 2.33) | 1.47 (0.97, 2.24) | 0.94 (0.64, 1.38) | 0.91 (0.58, 1.44) | 0.81 (0.38, 1.71) |
|  | Current drinker | 0.91 (0.71, 1.16) | 0.8 (0.69, 0.92) | 1.03 (0.91, 1.16) | 0.91 (0.8, 1.04) | 0.53 (0.42, 0.67) |
| Sleep duration | Less 6 hours | 1.05 (0.84, 1.31) | 1.07 (0.96, 1.2) | 1.02 (0.92, 1.14) | 0.93 (0.83, 1.05) | 0.88 (0.75, 1.02) |
|  | 6-8 hours | Ref. | Ref. | Ref. | Ref. | Ref. |
|  | Over 8 hours | 0.74 (0.36, 1.51) | 0.69 (0.47, 1.02) | 0.8 (0.58, 1.11) | 1.19 (0.9, 1.57) | 0.97 (0.64, 1.45) |
| Biweekly physical activity | Less 30 min | Ref. | Ref. | Ref. | Ref. | Ref. |
|  | 30-60 min | 0.67 (0.53, 0.84) | 0.81 (0.72, 0.91) | 0.92 (0.82, 1.04) | 0.79 (0.71, 0.89) | 0.82 (0.7, 0.95) |
|  | 60-120 min | 0.49 (0.36, 0.65) | 0.65 (0.56, 0.76) | 0.83 (0.72, 0.96) | 0.68 (0.59, 0.78) | 0.58 (0.47, 0.7) |
|  | Over 120 min | 0.68 (0.47, 0.98) | 0.92 (0.76, 1.12) | 0.91 (0.76, 1.09) | 0.77 (0.63, 0.93) | 0.63 (0.46, 0.85) |
| Eat on time and in regular amounts | No | Ref. | Ref. | Ref. | Ref. | Ref. |
|  | Yes | 0.74 (0.6, 0.92) | 0.73 (0.65, 0.82) | 0.93 (0.83, 1.03) | 0.94 (0.84, 1.05) | 0.88 (0.76, 1.02) |
| Daily servings of rice or flour products | None or less than 1 serving a day | Ref. | Ref. | Ref. | Ref. | Ref. |
|  | At least 1 serving | 0.7 (0.44, 1.11) | 0.94 (0.73, 1.19) | 1.02 (0.8, 1.29) | 1.03 (0.78, 1.34) | 1.18 (0.85, 1.63) |
| Weekly servings of meat | None or less than 1 serving a week | Ref. | Ref. | Ref. | Ref. | Ref. |
|  | At least 1 serving | 1.42 (0.75, 2.68) | 1.46 (1.06, 2) | 0.86 (0.68, 1.09) | 0.95 (0.73, 1.25) | 0.83 (0.62, 1.12) |
| Daily servings of vegetables | None or less than ½ serving a day | Ref. | Ref. | Ref. | Ref. | Ref. |
|  | At least ½ serving | 1.05 (0.69, 1.6) | 1.11 (0.88, 1.4) | 0.95 (0.77, 1.17) | 0.97 (0.79, 1.21) | 1.02 (0.76, 1.37) |
| Daily servings of fruits | None or less than 1 serving a day | Ref. | Ref. | Ref. | Ref. | Ref. |
|  | At least 1 serving | 1.22 (0.96, 1.55) | 0.92 (0.82, 1.03) | 1.08 (0.96, 1.21) | 1.09 (0.98, 1.23) | 1.15 (0.98, 1.35) |

*The effects are estimated by hazard ratios and their 95% CIs

Table S7 Effect estimates from the co-exposure models for monthly PM_2.5_ exposure (per 1 μg/m^3^ increase) and all-day traffic noise exposure (per 1 dBA increase) of valid male participants

| Covariates | Category | Metabolic syndrome | Abdominal obesity | High blood pressure | High triglycerides | Low HDL cholesterol |
| --- | --- | --- | --- | --- | --- | --- |
| Monthly PM_2.5_ |  | 1.89 (1.65, 2.18) | 1.22 (1.13, 1.31) | 1.33 (1.24, 1.41) | 1.36 (1.27, 1.45) | 1.78 (1.59, 1.99) |
| Monthly L_den_ |  | 1.08 (1.03, 1.14) | 1.03 (1, 1.06) | 1.03 (1, 1.06) | 1.06 (1.04, 1.09) | 1.07 (1.03, 1.12) |
| Age | 20-39 years | Ref. | Ref. | Ref. | Ref. | Ref. |
|  | 40-49 years | 0.88 (0.66, 1.16) | 0.95 (0.81, 1.11) | 1.18 (1.02, 1.37) | 1.15 (1.01, 1.32) | 0.75 (0.6, 0.95) |
|  | 50-59 years | 0.72 (0.5, 1.03) | 0.74 (0.61, 0.92) | 1.21 (1.01, 1.44) | 0.91 (0.76, 1.09) | 0.46 (0.33, 0.65) |
|  | Over 60 years | 0.74 (0.44, 1.24) | 0.69 (0.51, 0.94) | 1.3 (1.03, 1.64) | 0.57 (0.41, 0.78) | 0.6 (0.37, 0.95) |
| Education level | High school diploma or less | Ref. | Ref. | Ref. | Ref. | Ref. |
|  | Bachelor degree | 0.73 (0.51, 1.04) | 0.93 (0.75, 1.16) | 0.92 (0.77, 1.1) | 0.98 (0.8, 1.19) | 0.82 (0.58, 1.15) |
|  | Master's degree or above | 0.69 (0.46, 1.03) | 0.79 (0.62, 1) | 0.83 (0.68, 1.02) | 0.98 (0.79, 1.22) | 0.74 (0.5, 1.08) |
| Marital status | Single | Ref. | Ref. | Ref. | Ref. | Ref. |
|  | Married/cohabitating | 1.24 (0.9, 1.69) | 1.36 (1.14, 1.61) | 0.93 (0.8, 1.08) | 1.3 (1.11, 1.51) | 1.31 (1.02, 1.69) |
|  | divorced | 1.29 (0.6, 2.77) | 1.09 (0.69, 1.7) | 0.78 (0.52, 1.17) | 1.35 (0.93, 1.98) | 1.15 (0.57, 2.34) |
|  | Widowed | 1.18 (0.27, 5.14) | 1.2 (0.48, 3.01) | 0.74 (0.4, 1.35) | 0.91 (0.37, 2.25) | 0 (0, Inf) |
| Family health history | No | Ref. | Ref. | Ref. | Ref. | Ref. |
|  | Yes | 1.13 (0.9, 1.42) | 1.13 (1, 1.28) | 1.1 (0.98, 1.23) | 1.06 (0.95, 1.19) | 0.83 (0.68, 1.01) |
| Smoking status | Never smoker | Ref. | Ref. | Ref. | Ref. | Ref. |
|  | Former smoker | 1.36 (0.96, 1.93) | 1.24 (1.02, 1.52) | 1.05 (0.88, 1.26) | 1.06 (0.88, 1.28) | 1.12 (0.79, 1.58) |
|  | Current smoker | 1.58 (1.2, 2.07) | 1.25 (1.07, 1.46) | 0.97 (0.84, 1.12) | 1.25 (1.09, 1.43) | 1.55 (1.23, 1.95) |
| Drinking status | Never drinker | Ref. | Ref. | Ref. | Ref. | Ref. |
|  | Former drinker | 1.07 (0.39, 2.92) | 1.29 (0.77, 2.17) | 1.14 (0.76, 1.7) | 0.96 (0.58, 1.57) | 0.87 (0.32, 2.34) |
|  | Current drinker | 0.95 (0.72, 1.24) | 0.87 (0.74, 1.01) | 1.08 (0.94, 1.23) | 0.96 (0.84, 1.1) | 0.57 (0.43, 0.74) |
| Sleep duration | Less 6 hours | 1.03 (0.79, 1.33) | 1.12 (0.97, 1.29) | 0.99 (0.86, 1.13) | 0.97 (0.85, 1.11) | 0.86 (0.68, 1.09) |
|  | 6-8 hours | Ref. | Ref. | Ref. | Ref. | Ref. |
|  | Over 8 hours | 0.51 (0.19, 1.39) | 0.61 (0.35, 1.05) | 0.87 (0.6, 1.28) | 1.09 (0.77, 1.53) | 0.56 (0.24, 1.26) |
| Biweekly physical activity | Less 30 min | Ref. | Ref. | Ref. | Ref. | Ref. |
|  | 30-60 min | 0.76 (0.58, 1) | 0.94 (0.8, 1.1) | 0.99 (0.86, 1.14) | 0.85 (0.74, 0.98) | 0.86 (0.68, 1.08) |
|  | 60-120 min | 0.49 (0.35, 0.7) | 0.75 (0.62, 0.9) | 0.88 (0.75, 1.04) | 0.67 (0.57, 0.79) | 0.47 (0.35, 0.64) |
|  | Over 120 min | 0.79 (0.53, 1.18) | 1.13 (0.9, 1.42) | 1.01 (0.82, 1.24) | 0.86 (0.7, 1.05) | 0.7 (0.48, 1.02) |
| Eat on time and in regular amounts | No | Ref. | Ref. | Ref. | Ref. | Ref. |
|  | Yes | 0.85 (0.66, 1.1) | 0.75 (0.65, 0.87) | 0.91 (0.8, 1.04) | 0.92 (0.81, 1.05) | 0.93 (0.74, 1.17) |
| Daily servings of rice or flour products | None or less than 1 serving a day | Ref. | Ref. | Ref. | Ref. | Ref. |
|  | At least 1 serving | 0.9 (0.46, 1.76) | 0.76 (0.54, 1.07) | 1.01 (0.73, 1.39) | 1.15 (0.78, 1.7) | 1.31 (0.67, 2.54) |
| Weekly servings of meat | None or less than 1 serving a week | Ref. | Ref. | Ref. | Ref. | Ref. |
|  | At least 1 serving | 1.46 (0.59, 3.59) | 1.12 (0.72, 1.74) | 0.73 (0.54, 0.98) | 1.06 (0.72, 1.55) | 0.8 (0.43, 1.47) |
| Daily servings of vegetables | None or less than ½ serving a day | Ref. | Ref. | Ref. | Ref. | Ref. |
|  | At least ½ serving | 0.9 (0.56, 1.44) | 1.11 (0.82, 1.5) | 1.04 (0.79, 1.36) | 0.92 (0.72, 1.18) | 0.93 (0.61, 1.4) |
| Daily servings of fruits | None or less than 1 serving a day | Ref. | Ref. | Ref. | Ref. | Ref. |
|  | At least 1 serving | 1.25 (0.95, 1.63) | 0.96 (0.83, 1.11) | 1.1 (0.97, 1.26) | 1.11 (0.97, 1.26) | 1.14 (0.91, 1.43) |

*The effects are estimated by hazard ratios and their 95% CIs

Table S8 Effect estimates from the co-exposure models for monthly PM_2.5_ exposure (per 1 μg/m^3^ increase) and nighttime traffic noise exposure (per 1 dBA increase) of valid male participants

| Covariates | Category | Metabolic syndrome | Abdominal obesity | High blood pressure | High triglycerides | Low HDL cholesterol |
| --- | --- | --- | --- | --- | --- | --- |
| Monthly PM_2.5_ |  | 1.88 (1.64, 2.16) | 1.22 (1.13, 1.31) | 1.33 (1.25, 1.41) | 1.36 (1.27, 1.45) | 1.77 (1.58, 1.98) |
| Monthly L_night_ |  | 1.05 (0.99, 1.12) | 1.01 (0.98, 1.05) | 1.02 (0.99, 1.05) | 1.06 (1.03, 1.09) | 1.06 (1.01, 1.12) |
| Age | 20-39 years | Ref. | Ref. | Ref. | Ref. | Ref. |
|  | 40-49 years | 0.86 (0.65, 1.14) | 0.94 (0.81, 1.1) | 1.18 (1.02, 1.36) | 1.14 (1, 1.31) | 0.75 (0.59, 0.95) |
|  | 50-59 years | 0.7 (0.49, 1) | 0.74 (0.6, 0.91) | 1.2 (1.01, 1.43) | 0.9 (0.75, 1.07) | 0.45 (0.32, 0.64) |
|  | Over 60 years | 0.71 (0.43, 1.19) | 0.68 (0.5, 0.92) | 1.28 (1.01, 1.63) | 0.56 (0.41, 0.77) | 0.58 (0.37, 0.93) |
| Education level | High school diploma or less | Ref. | Ref. | Ref. | Ref. | Ref. |
|  | Bachelor degree | 0.73 (0.51, 1.05) | 0.94 (0.75, 1.17) | 0.92 (0.77, 1.1) | 0.98 (0.81, 1.19) | 0.82 (0.58, 1.16) |
|  | Master's degree or above | 0.7 (0.46, 1.05) | 0.79 (0.62, 1.01) | 0.84 (0.68, 1.02) | 0.99 (0.8, 1.23) | 0.75 (0.51, 1.09) |
| Marital status | Single | Ref. | Ref. | Ref. | Ref. | Ref. |
|  | Married/cohabitating | 1.24 (0.91, 1.7) | 1.36 (1.14, 1.61) | 0.93 (0.8, 1.08) | 1.3 (1.12, 1.51) | 1.32 (1.02, 1.7) |
|  | divorced | 1.33 (0.62, 2.85) | 1.09 (0.7, 1.71) | 0.79 (0.53, 1.18) | 1.36 (0.93, 1.98) | 1.18 (0.58, 2.39) |
|  | Widowed | 1.22 (0.28, 5.3) | 1.21 (0.49, 3.04) | 0.74 (0.4, 1.36) | 0.92 (0.37, 2.27) | 0 (0, Inf) |
| Family health history | No | Ref. | Ref. | Ref. | Ref. | Ref. |
|  | Yes | 1.13 (0.89, 1.42) | 1.13 (1, 1.28) | 1.1 (0.98, 1.23) | 1.06 (0.95, 1.18) | 0.83 (0.68, 1) |
| Smoking status | Never smoker | Ref. | Ref. | Ref. | Ref. | Ref. |
|  | Former smoker | 1.38 (0.97, 1.95) | 1.25 (1.02, 1.52) | 1.06 (0.89, 1.27) | 1.07 (0.89, 1.28) | 1.13 (0.8, 1.59) |
|  | Current smoker | 1.58 (1.21, 2.08) | 1.25 (1.07, 1.46) | 0.97 (0.84, 1.12) | 1.25 (1.09, 1.43) | 1.55 (1.23, 1.96) |
| Drinking status | Never drinker | Ref. | Ref. | Ref. | Ref. | Ref. |
|  | Former drinker | 1.06 (0.39, 2.88) | 1.29 (0.77, 2.17) | 1.14 (0.76, 1.7) | 0.95 (0.57, 1.56) | 0.87 (0.32, 2.34) |
|  | Current drinker | 0.95 (0.73, 1.25) | 0.87 (0.74, 1.01) | 1.08 (0.94, 1.23) | 0.96 (0.84, 1.11) | 0.57 (0.43, 0.74) |
| Sleep duration | Less 6 hours | 1.03 (0.8, 1.34) | 1.12 (0.97, 1.29) | 0.99 (0.87, 1.13) | 0.97 (0.85, 1.11) | 0.87 (0.69, 1.1) |
|  | 6-8 hours | Ref. | Ref. | Ref. | Ref. | Ref. |
|  | Over 8 hours | 0.5 (0.19, 1.37) | 0.61 (0.35, 1.05) | 0.88 (0.6, 1.28) | 1.09 (0.77, 1.53) | 0.55 (0.24, 1.25) |
| Biweekly physical activity | Less 30 min | Ref. | Ref. | Ref. | Ref. | Ref. |
|  | 30-60 min | 0.75 (0.57, 0.99) | 0.94 (0.8, 1.09) | 0.99 (0.86, 1.14) | 0.85 (0.74, 0.97) | 0.86 (0.68, 1.08) |
|  | 60-120 min | 0.49 (0.35, 0.7) | 0.74 (0.62, 0.9) | 0.88 (0.75, 1.04) | 0.67 (0.57, 0.79) | 0.47 (0.35, 0.63) |
|  | Over 120 min | 0.79 (0.53, 1.18) | 1.13 (0.9, 1.42) | 1.01 (0.83, 1.24) | 0.86 (0.7, 1.06) | 0.7 (0.48, 1.02) |
| Eat on time and in regular amounts | No | Ref. | Ref. | Ref. | Ref. | Ref. |
|  | Yes | 0.86 (0.66, 1.1) | 0.75 (0.65, 0.87) | 0.91 (0.8, 1.03) | 0.93 (0.82, 1.05) | 0.93 (0.74, 1.17) |
| Daily servings of rice or flour products | None or less than 1 serving a day | Ref. | Ref. | Ref. | Ref. | Ref. |
|  | At least 1 serving | 0.91 (0.46, 1.79) | 0.76 (0.54, 1.07) | 1.01 (0.73, 1.39) | 1.15 (0.78, 1.71) | 1.31 (0.67, 2.55) |
| Weekly servings of meat | None or less than 1 serving a week | Ref. | Ref. | Ref. | Ref. | Ref. |
|  | At least 1 serving | 1.43 (0.58, 3.51) | 1.11 (0.72, 1.73) | 0.72 (0.53, 0.97) | 1.05 (0.71, 1.54) | 0.79 (0.43, 1.46) |
| Daily servings of vegetables | None or less than ½ serving a day | Ref. | Ref. | Ref. | Ref. | Ref. |
|  | At least ½ serving | 0.9 (0.56, 1.45) | 1.11 (0.82, 1.51) | 1.05 (0.8, 1.37) | 0.92 (0.72, 1.18) | 0.93 (0.62, 1.41) |
| Daily servings of fruits | None or less than 1 serving a day | Ref. | Ref. | Ref. | Ref. | Ref. |
|  | At least 1 serving | 1.26 (0.96, 1.65) | 0.96 (0.83, 1.11) | 1.11 (0.97, 1.27) | 1.11 (0.97, 1.26) | 1.14 (0.91, 1.44) |

*The effects are estimated by hazard ratios and their 95% CIs

Table S9 Effect estimates from the co-exposure models with interaction terms for monthly PM_2.5_ exposure (per 1 μg/m^3^ increase) and all-day traffic noise exposure (per 1 dBA increase) of valid male participants

| Covariates | Category | Metabolic syndrome | Abdominal obesity | High blood pressure | High triglycerides | Low HDL cholesterol |
| --- | --- | --- | --- | --- | --- | --- |
| Monthly PM_2.5_ | PM_2.5_ ≥ 19.73 μg/m^3^ | 3.68 (2.27, 5.98) | 2.33 (1.83, 2.96) | 2.32 (1.88, 2.87) | 3.06 (2.43, 3.86) | 2.81 (1.87, 4.23) |
|  | PM_2.5_ < 19.73 μg/m^3^ | Ref. | Ref. | Ref. | Ref. | Ref. |
| Monthly L_den_ | L_den_ ≥ 67.91 dBA | 1.59 (1.02, 2.48) | 1.5 (1.2, 1.88) | 1.37 (1.12, 1.68) | 1.85 (1.48, 2.3) | 1.97 (1.38, 2.81) |
|  | L_den_ < 67.91 dBA | Ref. | Ref. | Ref. | Ref. | Ref. |
| PM_2.5_ × L_den_ | PM_2.5_ ≥ and L_den_ ≥ | 1.54 (0.89, 2.67) | 1.15 (0.86, 1.52) | 1.29 (1, 1.66) | 0.88 (0.67, 1.15) | 1.12 (0.71, 1.78) |
|  | At least one at low level | Ref. | Ref. | Ref. | Ref. | Ref. |
| Age | 20-39 years | Ref. | Ref. | Ref. | Ref. | Ref. |
|  | 40-49 years | 0.82 (0.62, 1.1) | 0.92 (0.79, 1.08) | 1.17 (1.02, 1.36) | 1.14 (0.99, 1.3) | 0.76 (0.6, 0.96) |
|  | 50-59 years | 0.67 (0.46, 0.95) | 0.77 (0.62, 0.94) | 1.2 (1.01, 1.44) | 0.9 (0.75, 1.08) | 0.48 (0.34, 0.67) |
|  | Over 60 years | 0.64 (0.38, 1.07) | 0.7 (0.52, 0.95) | 1.37 (1.08, 1.73) | 0.6 (0.44, 0.82) | 0.68 (0.43, 1.08) |
| Education level | High school diploma or less | Ref. | Ref. | Ref. | Ref. | Ref. |
|  | Bachelor degree | 0.8 (0.56, 1.14) | 0.98 (0.78, 1.22) | 0.97 (0.81, 1.16) | 1.02 (0.84, 1.24) | 0.82 (0.58, 1.15) |
|  | Master's degree or above | 0.81 (0.54, 1.22) | 0.87 (0.68, 1.11) | 0.93 (0.76, 1.14) | 1.09 (0.88, 1.35) | 0.76 (0.52, 1.12) |
| Marital status | Single | Ref. | Ref. | Ref. | Ref. | Ref. |
|  | Married/cohabitating | 1.23 (0.9, 1.69) | 1.36 (1.15, 1.62) | 0.91 (0.78, 1.06) | 1.28 (1.1, 1.49) | 1.25 (0.97, 1.61) |
|  | divorced | 1.47 (0.68, 3.16) | 1.14 (0.72, 1.79) | 0.76 (0.51, 1.14) | 1.34 (0.92, 1.96) | 1.14 (0.56, 2.31) |
|  | Widowed | 1.69 (0.39, 7.35) | 1.36 (0.55, 3.4) | 0.69 (0.37, 1.26) | 1 (0.4, 2.47) | 0 (0, Inf) |
| Family health history | No | Ref. | Ref. | Ref. | Ref. | Ref. |
|  | Yes | 1.07 (0.85, 1.35) | 1.16 (1.02, 1.32) | 1.09 (0.98, 1.22) | 1.05 (0.94, 1.17) | 0.82 (0.68, 1) |
| Smoking status | Never smoker | Ref. | Ref. | Ref. | Ref. | Ref. |
|  | Former smoker | 1.34 (0.94, 1.9) | 1.22 (1, 1.49) | 1.04 (0.87, 1.25) | 1.05 (0.87, 1.27) | 1.08 (0.76, 1.52) |
|  | Current smoker | 1.44 (1.09, 1.89) | 1.2 (1.03, 1.4) | 0.92 (0.79, 1.06) | 1.16 (1.02, 1.33) | 1.49 (1.18, 1.89) |
| Drinking status | Never drinker | Ref. | Ref. | Ref. | Ref. | Ref. |
|  | Former drinker | 0.87 (0.32, 2.37) | 1.25 (0.74, 2.1) | 1.04 (0.69, 1.55) | 0.86 (0.52, 1.41) | 0.86 (0.32, 2.33) |
|  | Current drinker | 0.92 (0.7, 1.21) | 0.86 (0.74, 1.01) | 1.06 (0.93, 1.21) | 0.96 (0.84, 1.1) | 0.59 (0.45, 0.77) |
| Sleep duration | Less 6 hours | 1.1 (0.85, 1.43) | 1.14 (0.99, 1.32) | 1.04 (0.91, 1.19) | 1 (0.87, 1.14) | 0.88 (0.69, 1.11) |
|  | 6-8 hours | Ref. | Ref. | Ref. | Ref. | Ref. |
|  | Over 8 hours | 0.54 (0.2, 1.47) | 0.65 (0.37, 1.13) | 0.98 (0.67, 1.44) | 1.14 (0.81, 1.6) | 0.63 (0.28, 1.44) |
| Biweekly physical activity | Less 30 min | Ref. | Ref. | Ref. | Ref. | Ref. |
|  | 30-60 min | 0.76 (0.58, 0.99) | 0.94 (0.81, 1.1) | 0.98 (0.85, 1.13) | 0.85 (0.74, 0.98) | 0.85 (0.68, 1.07) |
|  | 60-120 min | 0.5 (0.35, 0.7) | 0.78 (0.65, 0.95) | 0.88 (0.74, 1.04) | 0.69 (0.59, 0.82) | 0.48 (0.35, 0.65) |
|  | Over 120 min | 0.88 (0.59, 1.31) | 1.18 (0.94, 1.48) | 1 (0.82, 1.23) | 0.92 (0.75, 1.13) | 0.75 (0.52, 1.1) |
| Eat on time and in regular amounts | No | Ref. | Ref. | Ref. | Ref. | Ref. |
|  | Yes | 0.94 (0.72, 1.21) | 0.8 (0.69, 0.92) | 0.94 (0.82, 1.06) | 0.95 (0.84, 1.08) | 0.96 (0.77, 1.21) |
| Daily servings of rice or flour products | None or less than 1 serving a day | Ref. | Ref. | Ref. | Ref. | Ref. |
|  | At least 1 serving | 0.94 (0.48, 1.85) | 0.82 (0.58, 1.15) | 1.06 (0.77, 1.46) | 1.14 (0.77, 1.68) | 1.4 (0.72, 2.72) |
| Weekly servings of meat | None or less than 1 serving a week | Ref. | Ref. | Ref. | Ref. | Ref. |
|  | At least 1 serving | 1.29 (0.53, 3.17) | 1.14 (0.73, 1.76) | 0.77 (0.57, 1.05) | 1.08 (0.73, 1.58) | 0.84 (0.46, 1.54) |
| Daily servings of vegetables | None or less than ½ serving a day | Ref. | Ref. | Ref. | Ref. | Ref. |
|  | At least ½ serving | 0.97 (0.61, 1.56) | 1.09 (0.8, 1.47) | 1.04 (0.8, 1.36) | 0.92 (0.72, 1.18) | 0.91 (0.6, 1.38) |
| Daily servings of fruits | None or less than 1 serving a day | Ref. | Ref. | Ref. | Ref. | Ref. |
|  | At least 1 serving | 1.2 (0.92, 1.58) | 0.93 (0.81, 1.08) | 1.07 (0.94, 1.23) | 1.09 (0.95, 1.24) | 1.12 (0.89, 1.41) |

*The effects are estimated by hazard ratios and their 95% CIs

Table S10 Effect estimates from the co-exposure models with interaction terms for monthly PM_2.5_ exposure (per 1 μg/m^3^ increase) and nighttime traffic noise exposure (per 1 dBA increase) of valid male participants

| Covariates | Category | Metabolic syndrome | Abdominal obesity | High blood pressure | High triglycerides | Low HDL cholesterol |
| --- | --- | --- | --- | --- | --- | --- |
| Monthly PM_2.5_ | PM_2.5_ ≥ 19.73 μg/m^3^ | 3.81 (2.52 ,5.76) | 2.48 (1.98 ,3.11) | 2.51 (2.06 ,3.07) | 3.04 (2.46 ,3.77) | 2.86 (2 ,4.09) |
|  | PM_2.5_ < 19.73 μg/m^3^ | Ref. | Ref. | Ref. | Ref. | Ref. |
| Monthly L_night_ | L_night_ ≥ 62.33 dBA | 1.34 (0.9 ,2.02) | 1.52 (1.22 ,1.88) | 1.41 (1.16 ,1.72) | 1.72 (1.4 ,2.12) | 1.66 (1.2 ,2.3) |
|  | L_night_ < 62.33 dBA | Ref. | Ref. | Ref. | Ref. | Ref. |
| PM_2.5_ × L_night_ | PM_2.5_ ≥ and L_night_ ≥ | 1.55 (0.94 ,2.55) | 1.05 (0.8 ,1.37) | 1.16 (0.9 ,1.48) | 0.89 (0.69 ,1.14) | 1.09 (0.72 ,1.67) |
|  | At least one at low level | Ref. | Ref. | Ref. | Ref. | Ref. |
| Age | 20-39 years | Ref. | Ref. | Ref. | Ref. | Ref. |
|  | 40-49 years | 0.83 (0.63 ,1.1) | 0.93 (0.8 ,1.09) | 1.19 (1.03 ,1.38) | 1.15 (1.01 ,1.31) | 0.76 (0.6 ,0.97) |
|  | 50-59 years | 0.66 (0.46 ,0.94) | 0.76 (0.62 ,0.93) | 1.22 (1.03 ,1.46) | 0.92 (0.77 ,1.09) | 0.47 (0.34 ,0.67) |
|  | Over 60 years | 0.67 (0.4 ,1.12) | 0.7 (0.52 ,0.94) | 1.37 (1.08 ,1.73) | 0.59 (0.43 ,0.81) | 0.67 (0.42 ,1.06) |
| Education level | High school diploma or less | Ref. | Ref. | Ref. | Ref. | Ref. |
|  | Bachelor degree | 0.77 (0.54 ,1.1) | 0.95 (0.76 ,1.18) | 0.96 (0.8 ,1.15) | 1.02 (0.84 ,1.24) | 0.82 (0.58 ,1.15) |
|  | Master's degree or above | 0.78 (0.52 ,1.17) | 0.84 (0.66 ,1.07) | 0.92 (0.75 ,1.13) | 1.08 (0.87 ,1.34) | 0.75 (0.51 ,1.11) |
| Marital status | Single | Ref. | Ref. | Ref. | Ref. | Ref. |
|  | Married/cohabitating | 1.25 (0.91 ,1.71) | 1.36 (1.14 ,1.61) | 0.91 (0.78 ,1.06) | 1.28 (1.1 ,1.49) | 1.26 (0.97 ,1.62) |
|  | divorced | 1.44 (0.67 ,3.09) | 1.13 (0.72 ,1.77) | 0.77 (0.51 ,1.15) | 1.37 (0.93 ,2) | 1.13 (0.56 ,2.29) |
|  | Widowed | 1.55 (0.36 ,6.75) | 1.35 (0.54 ,3.37) | 0.7 (0.38 ,1.28) | 0.98 (0.4 ,2.42) | 0 (0 ,Inf) |
| Family health history | No | Ref. | Ref. | Ref. | Ref. | Ref. |
|  | Yes | 1.08 (0.86 ,1.36) | 1.15 (1.01 ,1.31) | 1.09 (0.97 ,1.22) | 1.04 (0.93 ,1.17) | 0.82 (0.68 ,1) |
| Smoking status | Never smoker | Ref. | Ref. | Ref. | Ref. | Ref. |
|  | Former smoker | 1.37 (0.97 ,1.95) | 1.21 (0.99 ,1.48) | 1.02 (0.85 ,1.22) | 1.04 (0.86 ,1.25) | 1.09 (0.77 ,1.53) |
|  | Current smoker | 1.42 (1.08 ,1.87) | 1.18 (1.01 ,1.38) | 0.91 (0.79 ,1.05) | 1.15 (1 ,1.31) | 1.48 (1.17 ,1.88) |
| Drinking status | Never drinker | Ref. | Ref. | Ref. | Ref. | Ref. |
|  | Former drinker | 0.85 (0.31 ,2.34) | 1.22 (0.72 ,2.04) | 1.02 (0.68 ,1.53) | 0.88 (0.53 ,1.45) | 0.86 (0.32 ,2.32) |
|  | Current drinker | 0.94 (0.72 ,1.23) | 0.87 (0.74 ,1.01) | 1.07 (0.93 ,1.22) | 0.97 (0.84 ,1.11) | 0.59 (0.45 ,0.77) |
| Sleep duration | Less 6 hours | 1.12 (0.86 ,1.45) | 1.15 (0.99 ,1.32) | 1.02 (0.9 ,1.17) | 0.99 (0.87 ,1.13) | 0.88 (0.7 ,1.11) |
|  | 6-8 hours | Ref. | Ref. | Ref. | Ref. | Ref. |
|  | Over 8 hours | 0.54 (0.2 ,1.47) | 0.66 (0.38 ,1.15) | 0.96 (0.66 ,1.41) | 1.11 (0.79 ,1.56) | 0.61 (0.27 ,1.39) |
| Biweekly physical activity | Less 30 min | Ref. | Ref. | Ref. | Ref. | Ref. |
|  | 30-60 min | 0.76 (0.58 ,1) | 0.95 (0.81 ,1.11) | 0.98 (0.85 ,1.13) | 0.85 (0.74 ,0.97) | 0.85 (0.68 ,1.08) |
|  | 60-120 min | 0.5 (0.35 ,0.71) | 0.79 (0.65 ,0.95) | 0.88 (0.75 ,1.04) | 0.69 (0.59 ,0.82) | 0.48 (0.35 ,0.65) |
|  | Over 120 min | 0.89 (0.6 ,1.34) | 1.19 (0.95 ,1.49) | 1.01 (0.82 ,1.24) | 0.92 (0.75 ,1.13) | 0.76 (0.52 ,1.1) |
| Eat on time and in regular amounts | No | Ref. | Ref. | Ref. | Ref. | Ref. |
|  | Yes | 0.92 (0.72 ,1.19) | 0.79 (0.69 ,0.91) | 0.93 (0.82 ,1.06) | 0.95 (0.84 ,1.08) | 0.96 (0.76 ,1.21) |
| Daily servings of rice or flour products | None or less than 1 serving a day | Ref. | Ref. | Ref. | Ref. | Ref. |
|  | At least 1 serving | 0.94 (0.48 ,1.85) | 0.82 (0.58 ,1.15) | 1.01 (0.73 ,1.39) | 1.12 (0.76 ,1.66) | 1.42 (0.73 ,2.76) |
| Weekly servings of meat | None or less than 1 serving a week | Ref. | Ref. | Ref. | Ref. | Ref. |
|  | At least 1 serving | 1.29 (0.53 ,3.18) | 1.11 (0.72 ,1.72) | 0.78 (0.58 ,1.06) | 1.07 (0.73 ,1.57) | 0.82 (0.45 ,1.51) |
| Daily servings of vegetables | None or less than ½ serving a day | Ref. | Ref. | Ref. | Ref. | Ref. |
|  | At least ½ serving | 0.93 (0.58 ,1.5) | 1.08 (0.8 ,1.47) | 1.04 (0.79 ,1.36) | 0.91 (0.71 ,1.17) | 0.89 (0.59 ,1.34) |
| Daily servings of fruits | None or less than 1 serving a day | Ref. | Ref. | Ref. | Ref. | Ref. |
|  | At least 1 serving | 1.24 (0.94 ,1.62) | 0.94 (0.82 ,1.09) | 1.09 (0.96 ,1.25) | 1.11 (0.97 ,1.26) | 1.15 (0.92 ,1.45) |

*The effects are estimated by hazard ratios and their 95% CIs

Table S11 Effect estimates from the co-exposure models for monthly PM_2.5_ exposure (per 1 μg/m^3^ increase) and all-day traffic noise exposure (per 1 dBA increase) of valid female participants

| Covariates | Category | Metabolic syndrome | Abdominal obesity | High blood pressure | High triglycerides | Low HDL cholesterol |
| --- | --- | --- | --- | --- | --- | --- |
| Monthly PM_2.5_ |  | 1.86 (1.45, 2.4) | 1.54 (1.39, 1.7) | 1.49 (1.33, 1.66) | 2.14 (1.9, 2.41) | 1.93 (1.74, 2.14) |
| Monthly L_den_ |  | 1.18 (1.08, 1.29) | 1.1 (1.05, 1.14) | 1.08 (1.03, 1.13) | 1.08 (1.03, 1.14) | 1.11 (1.06, 1.16) |
| Age | 20-39 years | Ref. | Ref. | Ref. | Ref. | Ref. |
|  | 40-49 years | 2.22 (1.29, 3.81) | 0.91 (0.74, 1.13) | 2.09 (1.58, 2.76) | 1.14 (0.88, 1.47) | 0.99 (0.8, 1.22) |
|  | 50-59 years | 2.64 (1.41, 4.94) | 0.97 (0.75, 1.26) | 3.61 (2.67, 4.89) | 1.59 (1.18, 2.14) | 0.92 (0.69, 1.22) |
|  | Over 60 years | 2.78 (1.23, 6.29) | 1.29 (0.91, 1.81) | 5.25 (3.7, 7.46) | 1.53 (1.01, 2.31) | 0.86 (0.56, 1.31) |
| Education level | High school diploma or less | Ref. | Ref. | Ref. | Ref. | Ref. |
|  | Bachelor degree | 0.63 (0.39, 1.03) | 0.77 (0.61, 0.98) | 0.72 (0.57, 0.9) | 0.68 (0.53, 0.88) | 0.75 (0.58, 0.96) |
|  | Master's degree or above | 0.34 (0.15, 0.78) | 0.61 (0.44, 0.84) | 0.64 (0.46, 0.9) | 0.64 (0.45, 0.91) | 0.87 (0.63, 1.2) |
| Marital status | Single | Ref. | Ref. | Ref. | Ref. | Ref. |
|  | Married/cohabitating | 1.19 (0.7, 2.02) | 1.24 (1, 1.52) | 0.92 (0.71, 1.17) | 1.24 (0.95, 1.61) | 1 (0.81, 1.24) |
|  | divorced | 0.79 (0.26, 2.39) | 1.24 (0.8, 1.93) | 0.84 (0.54, 1.32) | 1.6 (1.03, 2.48) | 1 (0.63, 1.59) |
|  | Widowed | 2.04 (0.76, 5.46) | 1.84 (1.15, 2.94) | 1.36 (0.89, 2.07) | 1.61 (0.88, 2.92) | 1.3 (0.71, 2.38) |
| Family health history | No | Ref. | Ref. | Ref. | Ref. | Ref. |
|  | Yes | 1.12 (0.75, 1.65) | 1.09 (0.92, 1.29) | 1.08 (0.9, 1.3) | 1.16 (0.95, 1.42) | 1.04 (0.87, 1.24) |
| Smoking status | Never smoker | Ref. | Ref. | Ref. | Ref. | Ref. |
|  | Former smoker | 1.86 (0.51, 6.81) | 1.82 (1.04, 3.21) | 1.56 (0.79, 3.05) | 1.75 (0.94, 3.27) | 1.2 (0.61, 2.36) |
|  | Current smoker | 0.39 (0.12, 1.29) | 0.77 (0.51, 1.14) | 0.42 (0.22, 0.8) | 1.38 (0.9, 2.12) | 1.09 (0.72, 1.65) |
| Drinking status | Never drinker | Ref. | Ref. | Ref. | Ref. | Ref. |
|  | Former drinker | 0.98 (0.1, 9.17) | 1.95 (0.89, 4.3) | 0.6 (0.14, 2.59) | 0.92 (0.28, 3) | 0.75 (0.23, 2.39) |
|  | Current drinker | 0.8 (0.39, 1.67) | 0.57 (0.39, 0.82) | 1.13 (0.77, 1.65) | 0.71 (0.48, 1.06) | 0.47 (0.31, 0.72) |
| Sleep duration | Less 6 hours | 1.04 (0.68, 1.59) | 1 (0.83, 1.2) | 1.06 (0.87, 1.29) | 0.78 (0.62, 0.99) | 0.88 (0.72, 1.08) |
|  | 6-8 hours | Ref. | Ref. | Ref. | Ref. | Ref. |
|  | Over 8 hours | 1.29 (0.47, 3.59) | 0.84 (0.49, 1.43) | 0.69 (0.36, 1.29) | 1.62 (1.02, 2.59) | 1.29 (0.8, 2.08) |
| Biweekly physical activity | Less 30 min | Ref. | Ref. | Ref. | Ref. | Ref. |
|  | 30-60 min | 0.5 (0.32, 0.77) | 0.66 (0.55, 0.8) | 0.88 (0.71, 1.09) | 0.69 (0.55, 0.87) | 0.78 (0.64, 0.96) |
|  | 60-120 min | 0.45 (0.26, 0.78) | 0.54 (0.42, 0.69) | 0.76 (0.58, 0.98) | 0.73 (0.55, 0.95) | 0.68 (0.52, 0.88) |
|  | Over 120 min | 0.25 (0.06, 1.05) | 0.55 (0.34, 0.88) | 0.71 (0.45, 1.11) | 0.41 (0.22, 0.73) | 0.49 (0.29, 0.82) |
| Eat on time and in regular amounts | No | Ref. | Ref. | Ref. | Ref. | Ref. |
|  | Yes | 0.57 (0.38, 0.86) | 0.7 (0.59, 0.84) | 1.02 (0.82, 1.27) | 1 (0.8, 1.25) | 0.84 (0.69, 1.02) |
| Daily servings of rice or flour products | None or less than 1 serving a day | Ref. | Ref. | Ref. | Ref. | Ref. |
|  | At least 1 serving | 0.5 (0.26, 0.95) | 1.19 (0.84, 1.68) | 1.07 (0.74, 1.56) | 0.97 (0.66, 1.42) | 1.15 (0.79, 1.67) |
| Weekly servings of meat | None or less than 1 serving a week | Ref. | Ref. | Ref. | Ref. | Ref. |
|  | At least 1 serving | 1.65 (0.65, 4.17) | 1.93 (1.22, 3.07) | 1.09 (0.75, 1.6) | 0.96 (0.65, 1.42) | 0.87 (0.62, 1.23) |
| Daily servings of vegetables | None or less than ½ serving a day | Ref. | Ref. | Ref. | Ref. | Ref. |
|  | At least ½ serving | 1.38 (0.54, 3.48) | 1.05 (0.74, 1.51) | 0.67 (0.47, 0.96) | 1.13 (0.73, 1.75) | 1.09 (0.71, 1.65) |
| Daily servings of fruits | None or less than 1 serving a day | Ref. | Ref. | Ref. | Ref. | Ref. |
|  | At least 1 serving | 1.14 (0.68, 1.91) | 0.85 (0.7, 1.04) | 1 (0.78, 1.29) | 1.06 (0.82, 1.36) | 1.2 (0.96, 1.5) |

*The effects are estimated by hazard ratios and their 95% CIs

Table S12 Effect estimates from the co-exposure models for monthly PM_2.5_ exposure (per 1 μg/m^3^ increase) and nighttime traffic noise exposure (per 1 dBA increase) of valid female participants

| Covariates | Category | Metabolic syndrome | Abdominal obesity | High blood pressure | High triglycerides | Low HDL cholesterol |
| --- | --- | --- | --- | --- | --- | --- |
| Monthly PM_2.5_ |  | 1.87 (1.46, 2.4) | 1.54 (1.39, 1.69) | 1.49 (1.33, 1.67) | 2.14 (1.9, 2.41) | 1.92 (1.73, 2.13) |
| Monthly L_night_ |  | 1.14 (1.03, 1.27) | 1.09 (1.04, 1.14) | 1.06 (1, 1.12) | 1.07 (1.01, 1.13) | 1.1 (1.05, 1.15) |
| Age | 20-39 years | Ref. | Ref. | Ref. | Ref. | Ref. |
|  | 40-49 years | 2.21 (1.29, 3.8) | 0.91 (0.74, 1.12) | 2.09 (1.58, 2.76) | 1.13 (0.87, 1.46) | 0.99 (0.8, 1.22) |
|  | 50-59 years | 2.59 (1.39, 4.85) | 0.97 (0.75, 1.26) | 3.59 (2.65, 4.86) | 1.58 (1.17, 2.12) | 0.91 (0.68, 1.21) |
|  | Over 60 years | 2.78 (1.23, 6.27) | 1.28 (0.91, 1.81) | 5.25 (3.69, 7.45) | 1.52 (1, 2.3) | 0.87 (0.57, 1.32) |
| Education level | High school diploma or less | Ref. | Ref. | Ref. | Ref. | Ref. |
|  | Bachelor degree | 0.64 (0.4, 1.04) | 0.78 (0.62, 0.98) | 0.72 (0.57, 0.91) | 0.69 (0.54, 0.89) | 0.75 (0.58, 0.97) |
|  | Master's degree or above | 0.34 (0.15, 0.79) | 0.61 (0.45, 0.84) | 0.64 (0.46, 0.9) | 0.64 (0.45, 0.92) | 0.88 (0.64, 1.21) |
| Marital status | Single | Ref. | Ref. | Ref. | Ref. | Ref. |
|  | Married/cohabitating | 1.2 (0.7, 2.03) | 1.24 (1.01, 1.52) | 0.91 (0.71, 1.17) | 1.24 (0.95, 1.61) | 1 (0.81, 1.23) |
|  | divorced | 0.8 (0.27, 2.42) | 1.27 (0.82, 1.96) | 0.84 (0.54, 1.33) | 1.61 (1.04, 2.49) | 1.01 (0.63, 1.6) |
|  | Widowed | 2.09 (0.78, 5.6) | 1.85 (1.16, 2.96) | 1.35 (0.89, 2.06) | 1.62 (0.89, 2.94) | 1.3 (0.71, 2.38) |
| Family health history | No | Ref. | Ref. | Ref. | Ref. | Ref. |
|  | Yes | 1.11 (0.75, 1.65) | 1.09 (0.92, 1.29) | 1.08 (0.9, 1.3) | 1.17 (0.96, 1.42) | 1.04 (0.87, 1.24) |
| Smoking status | Never smoker | Ref. | Ref. | Ref. | Ref. | Ref. |
|  | Former smoker | 1.8 (0.5, 6.51) | 1.81 (1.03, 3.18) | 1.53 (0.78, 3) | 1.72 (0.92, 3.21) | 1.18 (0.6, 2.33) |
|  | Current smoker | 0.4 (0.12, 1.33) | 0.77 (0.52, 1.15) | 0.42 (0.22, 0.8) | 1.37 (0.89, 2.11) | 1.1 (0.73, 1.66) |
| Drinking status | Never drinker | Ref. | Ref. | Ref. | Ref. | Ref. |
|  | Former drinker | 1.04 (0.11, 9.56) | 1.98 (0.9, 4.34) | 0.62 (0.14, 2.64) | 0.94 (0.29, 3.07) | 0.76 (0.24, 2.44) |
|  | Current drinker | 0.83 (0.4, 1.73) | 0.57 (0.4, 0.83) | 1.13 (0.77, 1.65) | 0.72 (0.49, 1.07) | 0.47 (0.31, 0.72) |
| Sleep duration | Less 6 hours | 1.03 (0.67, 1.58) | 1 (0.83, 1.2) | 1.06 (0.86, 1.29) | 0.78 (0.62, 0.99) | 0.88 (0.72, 1.08) |
|  | 6-8 hours | Ref. | Ref. | Ref. | Ref. | Ref. |
|  | Over 8 hours | 1.25 (0.45, 3.47) | 0.84 (0.49, 1.43) | 0.7 (0.37, 1.31) | 1.61 (1.01, 2.56) | 1.29 (0.8, 2.08) |
| Biweekly physical activity | Less 30 min | Ref. | Ref. | Ref. | Ref. | Ref. |
|  | 30-60 min | 0.5 (0.32, 0.77) | 0.66 (0.55, 0.8) | 0.88 (0.71, 1.09) | 0.69 (0.55, 0.87) | 0.78 (0.64, 0.96) |
|  | 60-120 min | 0.43 (0.25, 0.75) | 0.54 (0.42, 0.69) | 0.75 (0.58, 0.98) | 0.72 (0.55, 0.94) | 0.67 (0.52, 0.87) |
|  | Over 120 min | 0.25 (0.06, 1.04) | 0.56 (0.35, 0.89) | 0.71 (0.45, 1.12) | 0.41 (0.23, 0.74) | 0.49 (0.29, 0.82) |
| Eat on time and in regular amounts | No | Ref. | Ref. | Ref. | Ref. | Ref. |
|  | Yes | 0.55 (0.37, 0.83) | 0.7 (0.58, 0.83) | 1.01 (0.82, 1.26) | 0.99 (0.79, 1.23) | 0.84 (0.69, 1.02) |
| Daily servings of rice or flour products | None or less than 1 serving a day | Ref. | Ref. | Ref. | Ref. | Ref. |
|  | At least 1 serving | 0.5 (0.26, 0.96) | 1.2 (0.85, 1.69) | 1.07 (0.74, 1.56) | 0.97 (0.66, 1.41) | 1.15 (0.79, 1.68) |
| Weekly servings of meat | None or less than 1 serving a week | Ref. | Ref. | Ref. | Ref. | Ref. |
|  | At least 1 serving | 1.65 (0.65, 4.17) | 1.93 (1.22, 3.07) | 1.09 (0.75, 1.6) | 0.96 (0.65, 1.42) | 0.87 (0.62, 1.23) |
| Daily servings of vegetables | None or less than ½ serving a day | Ref. | Ref. | Ref. | Ref. | Ref. |
|  | At least ½ serving | 1.39 (0.55, 3.51) | 1.06 (0.74, 1.52) | 0.67 (0.47, 0.97) | 1.13 (0.73, 1.76) | 1.09 (0.72, 1.66) |
| Daily servings of fruits | None or less than 1 serving a day | Ref. | Ref. | Ref. | Ref. | Ref. |
|  | At least 1 serving | 1.13 (0.67, 1.89) | 0.85 (0.7, 1.03) | 1 (0.78, 1.28) | 1.06 (0.82, 1.36) | 1.2 (0.96, 1.5) |

*The effects are estimated by hazard ratios and their 95% CIs

Table S13 Effect estimates from the co-exposure models with interaction terms for monthly PM_2.5_ exposure (per 1 μg/m^3^ increase) and all-day traffic noise exposure (per 1 dBA increase) of valid female participants

| Covariates | Category | Metabolic syndrome | Abdominal obesity | High blood pressure | High triglycerides | Low HDL cholesterol |
| --- | --- | --- | --- | --- | --- | --- |
| Monthly PM_2.5_ | PM_2.5_ ≥ 19.83 μg/m^3^ | 7.87 (3.18, 19.48) | 3.12 (2.25, 4.32) | 3.78 (2.68, 5.33) | 4.55 (3.12, 6.62) | 2.5 (1.77, 3.53) |
|  | PM_2.5_ < 19.83 μg/m^3^ | Ref. | Ref. | Ref. | Ref. | Ref. |
| Monthly L_den_ | L_den_ ≥ 67.67 dBA | 4.07 (1.69, 9.83) | 2.12 (1.55, 2.9) | 2.1 (1.49, 2.96) | 2.07 (1.41, 3.03) | 2.46 (1.82, 3.32) |
|  | L_den_ < 67.67 dBA | Ref. | Ref. | Ref. | Ref. | Ref. |
| PM_2.5_ × L_den_ |  | 0.58 (0.21, 1.58) | 1.17 (0.8, 1.72) | 1 (0.66, 1.52) | 1.07 (0.68, 1.67) | 1.05 (0.7, 1.56) |
| Age | 20-39 years | Ref. | Ref. | Ref. | Ref. | Ref. |
|  | 40-49 years | 2.39 (1.39, 4.1) | 1 (0.81, 1.23) | 2.17 (1.64, 2.87) | 1.23 (0.95, 1.6) | 1.02 (0.82, 1.26) |
|  | 50-59 years | 2.78 (1.49, 5.18) | 1.04 (0.81, 1.35) | 3.56 (2.64, 4.81) | 1.72 (1.28, 2.31) | 0.93 (0.7, 1.23) |
|  | Over 60 years | 3.33 (1.49, 7.48) | 1.47 (1.04, 2.09) | 5.33 (3.74, 7.59) | 1.66 (1.09, 2.52) | 0.93 (0.61, 1.41) |
| Education level | High school diploma or less | Ref. | Ref. | Ref. | Ref. | Ref. |
|  | Bachelor degree | 0.7 (0.43, 1.12) | 0.85 (0.67, 1.07) | 0.78 (0.62, 0.97) | 0.69 (0.54, 0.89) | 0.74 (0.57, 0.95) |
|  | Master's degree or above | 0.41 (0.18, 0.94) | 0.73 (0.53, 1) | 0.8 (0.57, 1.11) | 0.68 (0.48, 0.98) | 0.88 (0.64, 1.22) |
| Marital status | Single | Ref. | Ref. | Ref. | Ref. | Ref. |
|  | Married/cohabitating | 1.16 (0.68, 1.97) | 1.19 (0.97, 1.47) | 0.9 (0.7, 1.16) | 1.15 (0.89, 1.5) | 1 (0.81, 1.24) |
|  | divorced | 0.91 (0.3, 2.77) | 1.31 (0.85, 2.03) | 0.88 (0.56, 1.39) | 1.52 (0.98, 2.36) | 1.07 (0.67, 1.71) |
|  | Widowed | 2.21 (0.85, 5.76) | 1.77 (1.11, 2.84) | 1.18 (0.77, 1.81) | 1.63 (0.9, 2.96) | 1.19 (0.65, 2.15) |
| Family health history | No | Ref. | Ref. | Ref. | Ref. | Ref. |
|  | Yes | 1.14 (0.77, 1.69) | 1.1 (0.93, 1.3) | 1.07 (0.89, 1.29) | 1.16 (0.95, 1.41) | 1.07 (0.9, 1.28) |
| Smoking status | Never smoker | Ref. | Ref. | Ref. | Ref. | Ref. |
|  | Former smoker | 2.15 (0.59, 7.87) | 1.9 (1.09, 3.32) | 1.66 (0.85, 3.24) | 1.75 (0.94, 3.25) | 1.07 (0.54, 2.1) |
|  | Current smoker | 0.38 (0.12, 1.26) | 0.79 (0.53, 1.17) | 0.41 (0.21, 0.78) | 1.41 (0.92, 2.16) | 1.07 (0.71, 1.62) |
| Drinking status | Never drinker | Ref. | Ref. | Ref. | Ref. | Ref. |
|  | Former drinker | 1.15 (0.12, 10.63) | 1.62 (0.75, 3.52) | 0.47 (0.11, 2.01) | 1.04 (0.32, 3.38) | 0.64 (0.2, 2.06) |
|  | Current drinker | 0.91 (0.44, 1.88) | 0.59 (0.41, 0.85) | 1.15 (0.78, 1.69) | 0.72 (0.49, 1.07) | 0.48 (0.31, 0.74) |
| Sleep duration | Less 6 hours | 1.09 (0.71, 1.68) | 1.03 (0.86, 1.24) | 1.1 (0.9, 1.34) | 0.81 (0.65, 1.03) | 0.91 (0.74, 1.11) |
|  | 6-8 hours | Ref. | Ref. | Ref. | Ref. | Ref. |
|  | Over 8 hours | 1.17 (0.41, 3.29) | 0.93 (0.54, 1.58) | 0.78 (0.41, 1.47) | 1.63 (1.02, 2.62) | 1.28 (0.79, 2.06) |
| Biweekly physical activity | Less 30 min | Ref. | Ref. | Ref. | Ref. | Ref. |
|  | 30-60 min | 0.51 (0.33, 0.79) | 0.68 (0.56, 0.82) | 0.91 (0.73, 1.12) | 0.69 (0.55, 0.86) | 0.78 (0.64, 0.96) |
|  | 60-120 min | 0.43 (0.25, 0.75) | 0.57 (0.44, 0.73) | 0.77 (0.6, 1) | 0.76 (0.58, 0.99) | 0.68 (0.52, 0.88) |
|  | Over 120 min | 0.26 (0.06, 1.07) | 0.55 (0.34, 0.88) | 0.67 (0.43, 1.06) | 0.43 (0.24, 0.77) | 0.54 (0.32, 0.91) |
| Eat on time and in regular amounts | No | Ref. | Ref. | Ref. | Ref. | Ref. |
|  | Yes | 0.53 (0.35, 0.79) | 0.69 (0.58, 0.82) | 0.99 (0.8, 1.24) | 1 (0.79, 1.25) | 0.82 (0.68, 1) |
| Daily servings of rice or flour products | None or less than 1 serving a day | Ref. | Ref. | Ref. | Ref. | Ref. |
|  | At least 1 serving | 0.53 (0.28, 1.01) | 1.26 (0.89, 1.79) | 1.14 (0.78, 1.66) | 0.98 (0.67, 1.43) | 1.24 (0.85, 1.8) |
| Weekly servings of meat | None or less than 1 serving a week | Ref. | Ref. | Ref. | Ref. | Ref. |
|  | At least 1 serving | 1.48 (0.58, 3.75) | 1.89 (1.19, 3) | 1.01 (0.69, 1.48) | 1 (0.68, 1.47) | 0.83 (0.59, 1.18) |
| Daily servings of vegetables | None or less than ½ serving a day | Ref. | Ref. | Ref. | Ref. | Ref. |
|  | At least ½ serving | 1.4 (0.55, 3.56) | 1.04 (0.73, 1.48) | 0.71 (0.49, 1.02) | 1.27 (0.82, 1.98) | 1.09 (0.72, 1.66) |
| Daily servings of fruits | None or less than 1 serving a day | Ref. | Ref. | Ref. | Ref. | Ref. |
|  | At least 1 serving | 1.15 (0.68, 1.93) | 0.89 (0.73, 1.08) | 1.01 (0.79, 1.29) | 1.02 (0.79, 1.31) | 1.21 (0.97, 1.51) |

*The effects are estimated by hazard ratios and their 95% CIs

Table S14 Effect estimates from the co-exposure models with interaction terms for monthly PM_2.5_ exposure (per 1 μg/m^3^ increase) and nighttime traffic noise exposure (per 1 dBA increase) of valid female participants

| Covariates | Category | Metabolic syndrome | Abdominal obesity | High blood pressure | High triglycerides | Low HDL cholesterol |
| --- | --- | --- | --- | --- | --- | --- |
| Monthly PM_2.5_ | PM_2.5_ ≥ 19.83 μg/m^3^ | 8.69 (3.95, 19.11) | 3.35 (2.46, 4.55) | 4.19 (3.02, 5.8) | 5.02 (3.51, 7.16) | 2.46 (1.79, 3.37) |
|  | PM_2.5_ < 19.83 μg/m^3^ | Ref. | Ref. | Ref. | Ref. | Ref. |
| Monthly L_night_ | L_night_ ≥ 62.18 dBA | 3.87 (1.75, 8.55) | 2.14 (1.58, 2.9) | 2.15 (1.54, 3.01) | 2.15 (1.48, 3.11) | 2.2 (1.66, 2.93) |
|  | L_night_ < 62.18 dBA | Ref. | Ref. | Ref. | Ref. | Ref. |
| PM_2.5_ × L_night_ |  | 0.46 (0.18, 1.15) | 1.07 (0.74, 1.55) | 0.83 (0.55, 1.24) | 0.92 (0.59, 1.43) | 1.06 (0.72, 1.54) |
| Age | 20-39 years | Ref. | Ref. | Ref. | Ref. | Ref. |
|  | 40-49 years | 2.33 (1.36, 4.01) | 0.98 (0.79, 1.21) | 2.11 (1.6, 2.79) | 1.2 (0.93, 1.55) | 1.02 (0.83, 1.27) |
|  | 50-59 years | 2.78 (1.48, 5.2) | 1.04 (0.8, 1.34) | 3.55 (2.63, 4.8) | 1.73 (1.29, 2.32) | 0.93 (0.7, 1.24) |
|  | Over 60 years | 3.41 (1.51, 7.72) | 1.48 (1.04, 2.11) | 5.28 (3.7, 7.53) | 1.67 (1.1, 2.53) | 0.94 (0.62, 1.43) |
| Education level | High school diploma or less | Ref. | Ref. | Ref. | Ref. | Ref. |
|  | Bachelor degree | 0.71 (0.44, 1.15) | 0.85 (0.67, 1.08) | 0.78 (0.62, 0.98) | 0.7 (0.54, 0.9) | 0.75 (0.58, 0.96) |
|  | Master's degree or above | 0.45 (0.2, 1.03) | 0.73 (0.53, 1.01) | 0.8 (0.57, 1.12) | 0.7 (0.49, 1) | 0.89 (0.65, 1.23) |
| Marital status | Single | Ref. | Ref. | Ref. | Ref. | Ref. |
|  | Married/cohabitating | 1.16 (0.68, 1.99) | 1.21 (0.98, 1.48) | 0.92 (0.71, 1.18) | 1.18 (0.91, 1.53) | 0.98 (0.8, 1.21) |
|  | divorced | 0.85 (0.28, 2.6) | 1.29 (0.83, 2.01) | 0.87 (0.55, 1.37) | 1.53 (0.99, 2.36) | 1.03 (0.64, 1.63) |
|  | Widowed | 2.24 (0.85, 5.86) | 1.73 (1.08, 2.78) | 1.21 (0.79, 1.85) | 1.63 (0.9, 2.96) | 1.19 (0.65, 2.16) |
| Family health history | No | Ref. | Ref. | Ref. | Ref. | Ref. |
|  | Yes | 1.12 (0.75, 1.66) | 1.1 (0.93, 1.3) | 1.06 (0.89, 1.28) | 1.16 (0.95, 1.41) | 1.05 (0.88, 1.26) |
| Smoking status | Never smoker | Ref. | Ref. | Ref. | Ref. | Ref. |
|  | Former smoker | 2.26 (0.62, 8.29) | 2.07 (1.18, 3.63) | 1.73 (0.88, 3.39) | 1.85 (0.99, 3.45) | 1.12 (0.57, 2.2) |
|  | Current smoker | 0.38 (0.12, 1.26) | 0.77 (0.52, 1.16) | 0.41 (0.21, 0.79) | 1.39 (0.91, 2.14) | 1.11 (0.74, 1.67) |
| Drinking status | Never drinker | Ref. | Ref. | Ref. | Ref. | Ref. |
|  | Former drinker | 1.14 (0.12, 10.7) | 1.51 (0.69, 3.3) | 0.47 (0.11, 2.03) | 1 (0.31, 3.25) | 0.63 (0.2, 2.02) |
|  | Current drinker | 0.93 (0.45, 1.92) | 0.59 (0.41, 0.86) | 1.15 (0.78, 1.68) | 0.75 (0.51, 1.1) | 0.48 (0.31, 0.73) |
| Sleep duration | Less 6 hours | 1.08 (0.7, 1.66) | 1.04 (0.86, 1.25) | 1.09 (0.89, 1.34) | 0.82 (0.65, 1.03) | 0.9 (0.73, 1.1) |
|  | 6-8 hours | Ref. | Ref. | Ref. | Ref. | Ref. |
|  | Over 8 hours | 1.24 (0.44, 3.47) | 0.89 (0.52, 1.53) | 0.75 (0.4, 1.42) | 1.63 (1.02, 2.62) | 1.33 (0.82, 2.14) |
| Biweekly physical activity | Less 30 min | Ref. | Ref. | Ref. | Ref. | Ref. |
|  | 30-60 min | 0.52 (0.34, 0.8) | 0.68 (0.57, 0.82) | 0.91 (0.73, 1.12) | 0.7 (0.56, 0.88) | 0.78 (0.64, 0.95) |
|  | 60-120 min | 0.43 (0.25, 0.75) | 0.57 (0.45, 0.73) | 0.77 (0.59, 1) | 0.76 (0.58, 1) | 0.67 (0.52, 0.87) |
|  | Over 120 min | 0.25 (0.06, 1.06) | 0.54 (0.34, 0.87) | 0.7 (0.44, 1.1) | 0.42 (0.23, 0.77) | 0.54 (0.32, 0.92) |
| Eat on time and in regular amounts | No | Ref. | Ref. | Ref. | Ref. | Ref. |
|  | Yes | 0.5 (0.33, 0.75) | 0.7 (0.59, 0.84) | 1 (0.8, 1.24) | 1 (0.8, 1.25) | 0.82 (0.68, 1) |
| Daily servings of rice or flour products | None or less than 1 serving a day | Ref. | Ref. | Ref. | Ref. | Ref. |
|  | At least 1 serving | 0.52 (0.27, 0.99) | 1.29 (0.91, 1.83) | 1.13 (0.78, 1.65) | 0.98 (0.67, 1.43) | 1.2 (0.83, 1.75) |
| Weekly servings of meat | None or less than 1 serving a week | Ref. | Ref. | Ref. | Ref. | Ref. |
|  | At least 1 serving | 1.63 (0.64, 4.14) | 1.89 (1.19, 3.01) | 1.04 (0.71, 1.52) | 1.01 (0.69, 1.5) | 0.85 (0.6, 1.2) |
| Daily servings of vegetables | None or less than ½ serving a day | Ref. | Ref. | Ref. | Ref. | Ref. |
|  | At least ½ serving | 1.47 (0.58, 3.72) | 1.06 (0.74, 1.51) | 0.71 (0.5, 1.02) | 1.28 (0.82, 1.98) | 1.12 (0.74, 1.71) |
| Daily servings of fruits | None or less than 1 serving a day | Ref. | Ref. | Ref. | Ref. | Ref. |
|  | At least 1 serving | 1.12 (0.67, 1.89) | 0.89 (0.73, 1.09) | 1.02 (0.8, 1.31) | 1.02 (0.79, 1.31) | 1.2 (0.96, 1.5) |

*The effects are estimated by hazard ratios and their 95% CIs
